# Integrative taxonomy of the genus *Pseudostegana* (Diptera, Drosophilidae) from China, with descriptions of eleven new species

**DOI:** 10.7717/peerj.5160

**Published:** 2018-09-05

**Authors:** Jinming Lu, Yuan Zhang, Hongwei Chen

**Affiliations:** Department of Entomology, South China Agricultural University, Guangzhou, Guangdong, China

**Keywords:** Taxonomy, Drosophilidae, Biodiversity, DNA, Morphology

## Abstract

The genus *Pseudostegana* (Okada, 1978) currently contains thirty-nine described species. A number of *Pseudostegana* were collected from the fieldwork in southwestern China from 2010 to 2017. Eleven new species were discovered and are described from southwestern China: *Pseudostegana alpina* Zhang & Chen, sp. nov.; *Pseudostegana amnicola* Zhang & Chen, sp. nov.; *Pseudostegana amoena* Zhang & Chen, sp. nov.; *Pseudostegana mailangang* Zhang & Chen, sp. nov.; *Pseudostegana meiduo* Zhang & Chen, sp. nov.; *Pseudostegana meiji* Zhang & Chen, sp. nov.; *Pseudostegana mystica* Zhang & Chen, sp. nov.; *Pseudostegana stictiptrata* Zhang & Chen, sp. nov.; *Pseudostegana stigmatptera* Zhang & Chen, sp. nov.; *Pseudostegana ximalaya* Zhang & Chen, sp. nov. and *Pseudostegana zhuoma* Zhang & Chen, sp. nov. A key to all Chinese *Pseudostegana* species based on morphological characters is provided. Two mitochondrial loci (*COI* and *ND2*) and one nuclear locus (*28S rRNA*) were sequenced for the *Pseudostegana* specimens, and Bayesian and RAxML concatenated analyses were run. Molecular species delimitation is performed using the distance-based automatic barcode gap discovery (ABGD) method. Molecular data support the morphological characteristics observed among these Chinese species and confirm the new species as being distinctly different.

## Introduction

The genus *Pseudostegana* (Okada, 1978) is widely distributed in tropical area from Oriental to Australasian regions. Adult flies of this genus are yellow to black in body color, with a body length of about two to four mm. They are usually found resting on fallen logs, tussocks or fruits beside a stream, flapping their wings slowly like butterflies (Li, Gao & Chen, 2010). Currently, a total of 39 *Pseudostegana* species have been described (Chen, Toda & Wang, 2005; Li, Gao & Chen, 2010): 17 spp. from Malaysia, 12 spp. from China, five spp. from the Philippines, three spp. from Papua New Guinea, two spp. from Indonesia and one sp. from Vietnam. Chen, Toda & Wang (2005) revised the *Pseudostegana* taxonomy using morphological characters, and proposed six species groups (the *atrofrons*, *javana*, *latiparma*, *grandipalpis*, *fleximediata* and *zonaria* groups) based on wing patterns and male genitalia. Among these six species groups, the *javana*, *latiparma*, *fleximediata* and *zonaria* groups have been found in Southern China (Chen, Toda & Wang, 2005; Li, Gao & Chen, 2010).

There are few phylogenetic studies concerning the *Pseudostegana* genus (Okada, 1978; Li et al., 2013). Okada (1978) conducted a taxometric analysis on subgeneric relationships of the genus *Stegana* using 11 morphological characters and recognized a clade composed of the genera *Pseudostegana* and *Parastegana* Okada, 1971, which was placed as the sister group to the rest of the genus *Stegana*. Li et al. (2013) reconstructed a molecular phylogeny of the East Asian species in the *Stegana* genus group and confirmed the sister relationship of *Parastegana* and *Pseudostegana*. The phylogeny of species groups within the genus is still little known.

The *COI* gene is a commonly used marker for the DNA barcode identification and is potentially useful for species discovery and identification (Hebert et al., 2003; Robe, Machado & Bartholomei-Santos, 2012; Madeira et al., 2016; Melbourne et al., 2017). Additional genetic markers on mitochondrial (*ND2*, *16S*, *COII*) and nuclear (*28S*, *ITS1*, *ITS2*) genome have been used alongside the *COI* fragment for species discrimination and phylogenetic analysis (Li et al., 2013; Boykin et al., 2014; Jürgenstein, Kurina & Põldmaa, 2015; Madeira et al., 2016; Zhang, Li & Chen, 2016; Wong et al., 2017; Yusseff-Vanegas & Agnarsson, 2017).

Our paper addresses the study of the *Pseudostegana* in Southwest China. Considering the recent diversity and the phylogenetic reorganization of Steganinae subfamily, we used an integrative taxonomy approach to delimit new species from the *Pseudostegana* genus. This improves the knowledge of global and East Asia biodiversity, and could give insights to the origin of the *Pseudostegana* genus.

## Materials and Methods

### Sample collection and morphological treatment

**Figure 1:**
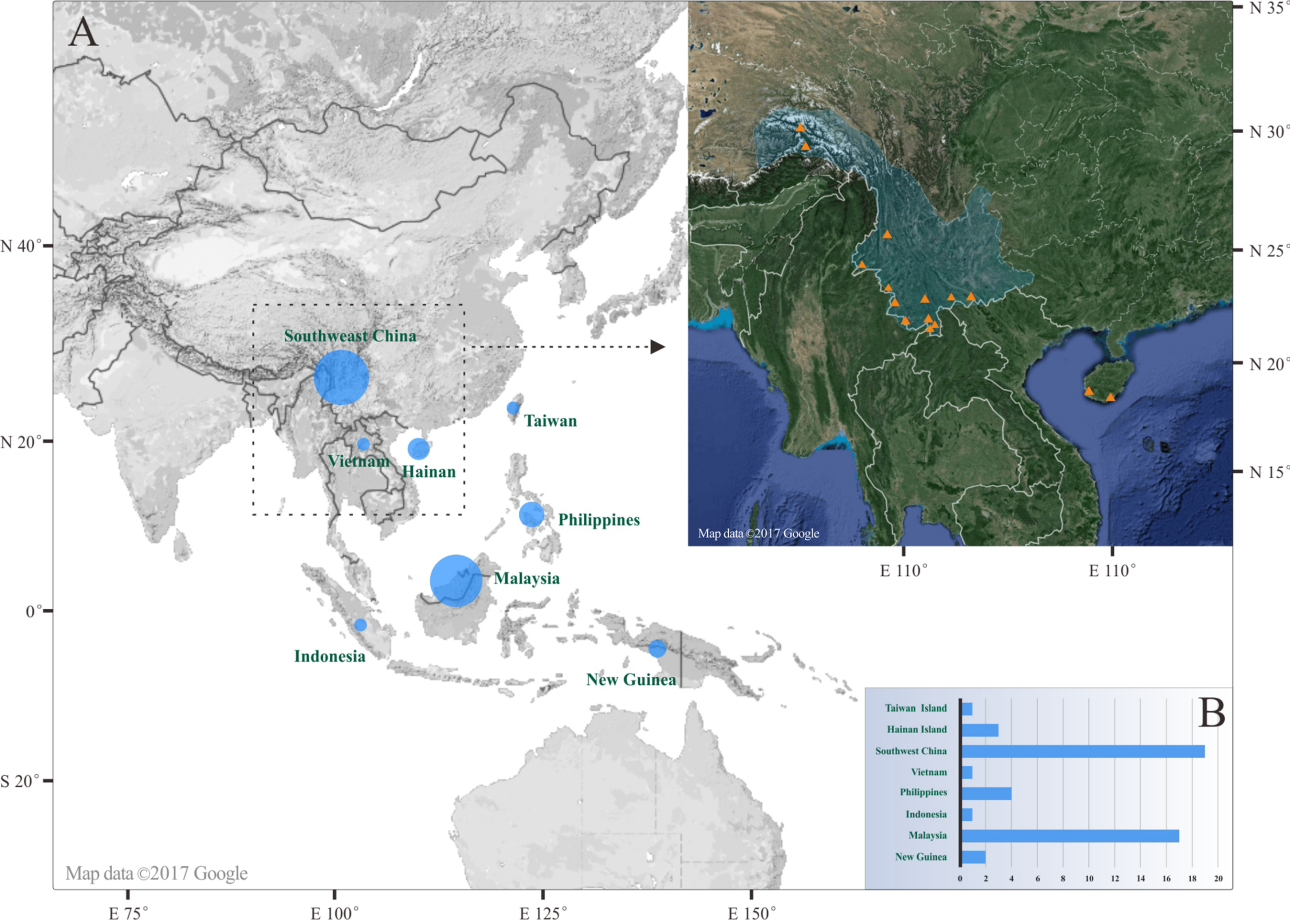
(A) Map of distribution of *Pseudostegana* species (map image © Google). (B) Size of blue circles and the length of blue bars is proportional to the number of *Pseudostegana* species. Orange triangles indicates the collection sites of the Chinese *Pseudostegana* in this study.

This study is based on material collected at several sites in China from 2010 to 2017 (Fig. 1). Specimens were collected by net-sweeping from tree trunks and then fixed in 75% ethanol. Male genitalia were removed from the abdomen, treated with 8% KOH for about 3–5 min and observed under the stereomicroscope (CX31; Olympus, Tokyo, Japan) for identification and drawing. Digital images were taken using a MD50 camera (Mshot, Guangzhou, China) mounted on the Olympus CX31 stereomicroscope. Some specimen was damaged during the extraction of the abdominal tissue, and thus we draw the morphological structure form the original abdomen. The female individuals were identified based on the description in Chen, Toda & Wang (2005) and Li, Gao & Chen (2010). The definitions of measurement abbreviations were followed as Zhang & Toda (1992) and Chen & Toda (2001) and listed in Table S1. The type specimens were deposited in the following institutions: Kunming Institute of Zoology (KIZ), Chinese Academy of Sciences, Kunming, China; Department of Entomology, South China Agricultural University (SCAU), Guangzhou, China.

The electronic version of this article in portable document format will represent a published work according to the International Commission on Zoological Nomenclature (ICZN), and hence the new names contained in the electronic version are effectively published under that code from the electronic edition alone. This published work and the nomenclatural acts it contains have been registered in ZooBank, the online registration system for the ICZN. The ZooBank LSIDs (Life Science Identifiers) can be resolved and the associated information viewed through any standard web browser by appending the LSID to the prefix http://zoobank.org/. The LSID for this publication is: urn:lsid:zoobank.org:pub:91F288F7-0083-4EBD-9B49-DF51048E9519. The online version of this work is archived and available from the following digital repositories: PeerJ, PubMed Central and CLOCKSS.

### DNA extraction, PCR and sequencing

Detailed information on samples used for molecular study is given in Table 1. Two *Parastegana* species, *Parastegana femorata* Duda, 1923 and *Parastegana brevivena* Chen & Zhang, 2007, are chosen as the outgroups in this study. Genomic DNA was extracted from the abdominal tissue of a single fly after the dissection of the genitalia using the TIANamp Genomic DNA kit (TIANGEN™, Beijing, China). DNA fragments of *COI*, *ND2* and *28S* were sequenced for each of the selected samples (Table 1). Target fragments were amplified and sequenced using published primers (Table S2). PCR products were sequenced at BGI (Beijing, China) by the ABI 3730 Genetic Analyzer (Applied Biosystems, Foster City, CA, USA) and were performed forward and reversed strand sequencing.

**Table 1:** Details of the samples from China used for molecular analyses in the study.

Genus	Species group	Species	Collection site	Latitude (°N)	Longitude (°E)	*COI*	*ND2*	*28S*
*Parastegana*		*femorata* Duda, 1923	Menglun, Mengla, Yunnan	21.6833	101.4167	KJ813937	KJ813971	KJ813904
		*brevivena* (Chen & Watabe, 2007)—1	Hesong, Menghai, Yunnan	21.8166	100.1000	KJ813938	KJ813972	KJ813905
		*brevivena* (Chen & Watabe, 2007)—2	Wuliangshan, Jingdong, Yunnan	24.5333	101.0167	KP981251	KY542068	KY542041
		*brevivena* (Chen & Watabe, 2007)—3	Wuliangshan, Jingdong, Yunnan	24.5333	101.0167	KP981252	KP981250	KY542040
*Pseudostegana*	*fleximediata*	*meiduo* Zhang & Chen, sp. nov.	Beibeng, Motuo, Xizang	29.3167	95.3333	KJ813939	KJ813973	KJ813906
	*javana*	*xanthoptera* Chen & Wang, 2005—1	Wangtianshu, Mengla, Yunnan	21.4667	101.6333	HQ842765*	HQ842786*	HQ842738*
		*xanthoptera* Chen & Wang, 2005—2	Wangtianshu, Mengla, Yunnan	21.4667	101.6333	KJ813940	KJ813974	KJ813907
		*xanthoptera* Chen & Wang, 2005—3	Yixiang, Pu’er, Yunnan	22.7833	101.0333	KJ813941	–	–
		*meiji* Zhang & Chen, sp. nov. —1	Muyiji Park, Ximeng, Yunnan	22.6208	99.5950	KJ813942	KJ813975	KJ813908
		*meiji* Zhang & Chen, sp. nov. —2	Muyiji Park, Ximeng, Yunnan	22.6208	99.5950	KY542055	KY542069	KY542042
		*meiji* Zhang & Chen, sp. nov. —3	Mengdong, Cangyuan, Yunnan	23.1689	99.2311	KY542056	KY542070	KY542043
		*stictiptrata* Zhang & Chen, sp. nov. —1	Hesong, Menghai, Yunnan	21.8166	100.1000	KJ813943	KJ813976	KJ813909
		*stictiptrata* Zhang & Chen, sp. nov. —2	Huanglianshan, Lvchun, Yunnan	22.8903	102.3167	KY542057	KY542071	KY542044
		*stigmatptera* Zhang & Chen, sp. nov. —1	Hesong, Menghai, Yunnan	21.8166	100.1000	KJ813944	KJ813977	KJ813910
		*stigmatptera* Zhang & Chen, sp. nov. —2	Hesong, Menghai, Yunnan	21.8166	100.1000	KJ813945	KJ813978	KJ813911
		*stigmatptera* Zhang & Chen, sp. nov. —3	Baihualing, Baoshan, Yunnan	25.4500	98.8667	KJ813946	–	–
	*latiparma*	*acutifoliolata* Li, Gao & Chen, 2010	Diaoluoshan, Lingshui, Hainan	18.6597	109.9025	KJ813947	KJ813979	KJ813912
		*angustifasciata* Chen & Wang, 2005—1	Wangtianshu, Mengla, Yunnan	21.4667	101.6333	HQ842766*	HQ842787*	HQ842739*
		*angustifasciata* Chen & Wang, 2005—2	Wangtianshu, Mengla, Yunnan	21.4667	101.6333	KJ813948	KJ813980	KJ813913
		*bifasciata* Chen & Wang, 2005—1	Wangtianshu, Mengla, Yunnan	21.4667	101.6333	KJ813949	KJ813981	KJ813914
		*bifasciata* Chen & Wang, 2005—2	Wangtianshu, Mengla, Yunnan	21.4667	101.6333	KJ813950	KJ813982	KJ813915
		*bilobata* Li, Gao & Chen, 2010—1	Yixiang, Pu’er, Yunnan	22.7833	101.0333	KJ813951	KJ813983	KJ813916
		*bilobata* Li, Gao & Chen, 2010—2	Muyiji Park, Ximeng, Yunnan	22.6208	99.5950	KY542058	KY542072	KY542045
		*minutipalpata* Li, Gao & Chen, 2010—1	Wangtianshu, Mengla, Yunnan	21.4667	101.6333	KJ813952	–	KJ813917
		*minutipalpata* Li, Gao & Chen, 2010—2	Wangtianshu, Mengla, Yunnan	21.4667	101.6333	KJ813953	KJ813984	KJ813918
		*minutipalpata* Li, Gao & Chen, 2010—3	Muyiji Park, Ximeng, Yunnan	22.6208	99.5950	KY542059	KY542073	KY542046
		*pallidemaculata* Chen & Wang, 2005	Fenshuiling, Jinping, Yunnan	22.9012	103.2302	KJ813954	KJ813985	KJ813919
		*alpina* Zhang & Chen, sp. nov.	Hesong, Menghai, Yunnan	21.8166	100.1000	KJ813955	KJ813986	KJ813920
		*amoena* Zhang & Chen, sp. nov.—1	Hesong, Menghai, Yunnan	21.8166	100.1000	KJ813956	KJ813987	KJ813921
		*amoena* Zhang & Chen, sp. nov.—2	Hesong, Menghai, Yunnan	21.8166	100.1000	KJ813957	KJ813988	KJ813922
		*amoena* Zhang & Chen, sp. nov.—3	Hesong, Menghai, Yunnan	21.8166	100.1000	KJ813958	KJ813989	KJ813923
		*ximalaya* Zhang & Chen, sp. nov.	Beibeng, Motuo, Xizang	29.3167	95.3333	KJ813959	KJ813990	KJ813924
		*zhuoma* Zhang & Chen, sp. nov.—1	Tongmai, Bomi, Xizang	30.1000	95.0833	KJ813960	KJ813991	KJ813925
		*zhuoma* Zhang & Chen, sp. nov.—2	Tongmai, Bomi, Xizang	30.1000	95.0833	KJ813961	KJ813992	KJ813926
	*zonaria*	*insularis* Li, Gao & Chen, 2010	Jianfengling, Ledong, Hainan	18.6800	108.8700	KJ813963	KJ813994	KJ813928
		*nitidifrons* Chen & Wang, 2005—1	Wangtianshu, Mengla, Yunnan	21.4667	101.6333	KJ813962	KJ813993	KJ813927
		*nitidifrons* Chen & Wang, 2005—2	Menglun, Mengla, Yunnan	21.6833	101.4167	HQ842767*	HQ842788*	HQ842740*
		*nitidifrons* Chen & Wang, 2005—3	Yixiang, Pu’er, Yunnan	22.7833	101.0333	KJ813964	KJ813995	KJ813929
		*nitidifrons* Chen & Wang, 2005—4	Mengyuan, Mengla, Yunnan	21.7914	101.3831	KY542063	KY542076	KY542049
		*silvana* Li, Gao & Chen, 2010—1	Diaoluoshan, Lingshui, Hainan	18.6597	109.9025	KJ813965	KJ813996	KJ813930
		*silvana* Li, Gao & Chen, 2010—2	Diaoluoshan, Lingshui, Hainan	18.6597	109.9025	–	KJ813997	KJ813931
		*amnicola* Zhang & Chen, sp. nov.—1	Beibeng, Motuo, Xizang	29.3167	95.3333	KJ813966	KJ813998	KJ813932
		*amnicola* Zhang & Chen, sp. nov.—2	Hesong, Menghai, Yunnan	21.8166	100.1000	KJ813967	KJ813999	KJ813933
		*amnicola* Zhang & Chen, sp. nov.—3	Baihualing, Baoshan, Yunnan	25.4500	98.8667	KJ813968	KJ814000	KJ813934
		*amnicola* Zhang & Chen, sp. nov.—4	Mengdong, Cangyuan, Yunnan	23.1689	99.2311	KY542064	KY542077	KY542050
		*mailangang* Zhang & Chen, sp. nov.—1	Wangtianshu, Mengla, Yunnan	21.4667	101.6333	KJ813969	KJ814001	KJ813935
		*mailangang* Zhang & Chen, sp. nov.—2	Guanlei, Mengla, Yunnan	21.6353	101.1722	KY542066	KY542080	KY542053
		*mystica* Zhang & Chen, sp. nov.	Beibeng, Motuo, Xizang	29.3167	95.3333	MH251912	–	–

**Note:**

* Sequences obtained in Li et al. (2013).

### Alignment and phylogenetic reconstruction

The obtained mitochondrial sequences were aligned with the ClustalW method implemented in MEGA 6.0 (Tamura et al., 2013) and they were translated into amino acid sequences to avoid the nuclear paralogous copies (Numts). In addition, the ratio between the number of synonymous substitutions per nonsynonymous sites (dN) and the number of synonymous substitutions per synonymous sites (dS) was assessed in MEGA 6.0 (Tamura et al., 2013) using Nei–Gojobori method (Nei & Gojobori, 1986). The “Q-INS-I” method in the online MAFFT software (http://mafft.cbrc.jp/alignment/server/) was applied for the alignment of *28S* data set.

Phylogenetic analyses were performed using maximum likelihood (ML) and Bayesian inference (BI) methods based on the combined data set of *COI*, *ND2* and *28S* segments (only the specimens with all three genes were employed). The combined alignment was partitioned into seven blocks, including six blocks for the first, second and third codon positions for the two mitochondrial coding genes, and one rRNA gene fragment. Then the partitioning schemes were searched for under PartitionFinder 1.1.1 (Lanfear et al., 2012) using the “greedy” algorithm with the Akaike’s Information Criteria (AIC) and corresponding optimal models were selected under the AIC using jModelTest v2.1.3 (Guindon & Gascuel, 2003; Darriba et al., 2012). The best partition scheme and substitution models were selected for BI or ML analysis: *COI* codon position 1 and 2 + *ND2* codon position 1 and 2 + *28S* − TIM+I+G; *COI* codon position 3 + *ND2* codon position 3 − GTR+G+I. BI was accessed in MrBayes 3.2.1 (Huelsenbeck & Ronquist, 2001; Ronquist & Huelsenbeck, 2003) and run on the CIPRES science gateway (http://www.phylo.org). Two independent runs with 20,000,000 generations were implemented in parallel and a sampling frequency of every 2,000 generations was employed. For each run the 4,000 early-phase samples were discarded as burn-in and the two runs were combined using LogCombiner (Drummond & Rambaut, 2007) to estimate a consensus tree. ML analysis was performed with RAxML GUI 1.3 (Silvestro & Michalak, 2012) with 20 random addition replicates. Of the models selected, the GTR+G+I model was used for the ML analysis. Reliability of the ML tree was assessed by thorough analysis for 1,000 bootstrap replications. A calculated posterior probability in the Bayesian tree ≥0.95 or a bootstrap support in the ML tree ≥70 was considered to indicate strong support for a given clade (Hillis & Bull, 1993; Erixon et al., 2003).

### Species delimitation

Pairwise genetic distances (Kimura-2-prameter) between taxa were calculated using MEGA 6.0 (Tamura et al., 2013). DNA-based species delimitations were tested using separate data sets of *COI*, *ND2* and *28S* with the automatic barcoding gap discovery (ABGD) method. The ABGD analyses were performed at the web interface (http://wwwabi.snv.jussieu.fr/public/abgd/, web version April 11, 2013), with a prior P that ranges from 0.005 to 0.1, and the simple distance model. This method statistically infers the DNA barcode gap in a single locus alignment, partitioning the data based on this gap in putative species (Puillandre et al., 2012).

## Results

### Phylogenetic analysis

The GenBank accession numbers for the obtained DNA sequences are shown in Table 1. The final sequence alignments length of *COI*, *ND2* and *28S* were 666 (252 variable, 225 parsimony informative), 1,047 (579 variable, 469 parsimony informative) and 1,002 (182 variable, 110 parsimony informative) bases long, respectively. Moreover, no strong evidence of Numts was found throughout our mitochondrial data, since neither stop-codons nor frameshifts were detected within the *COI* and *ND2* sequences. In addition, the dN/dS ratio analyses did not detect any recent *COI* or *ND2* pseudogene.

**Figure 2:**
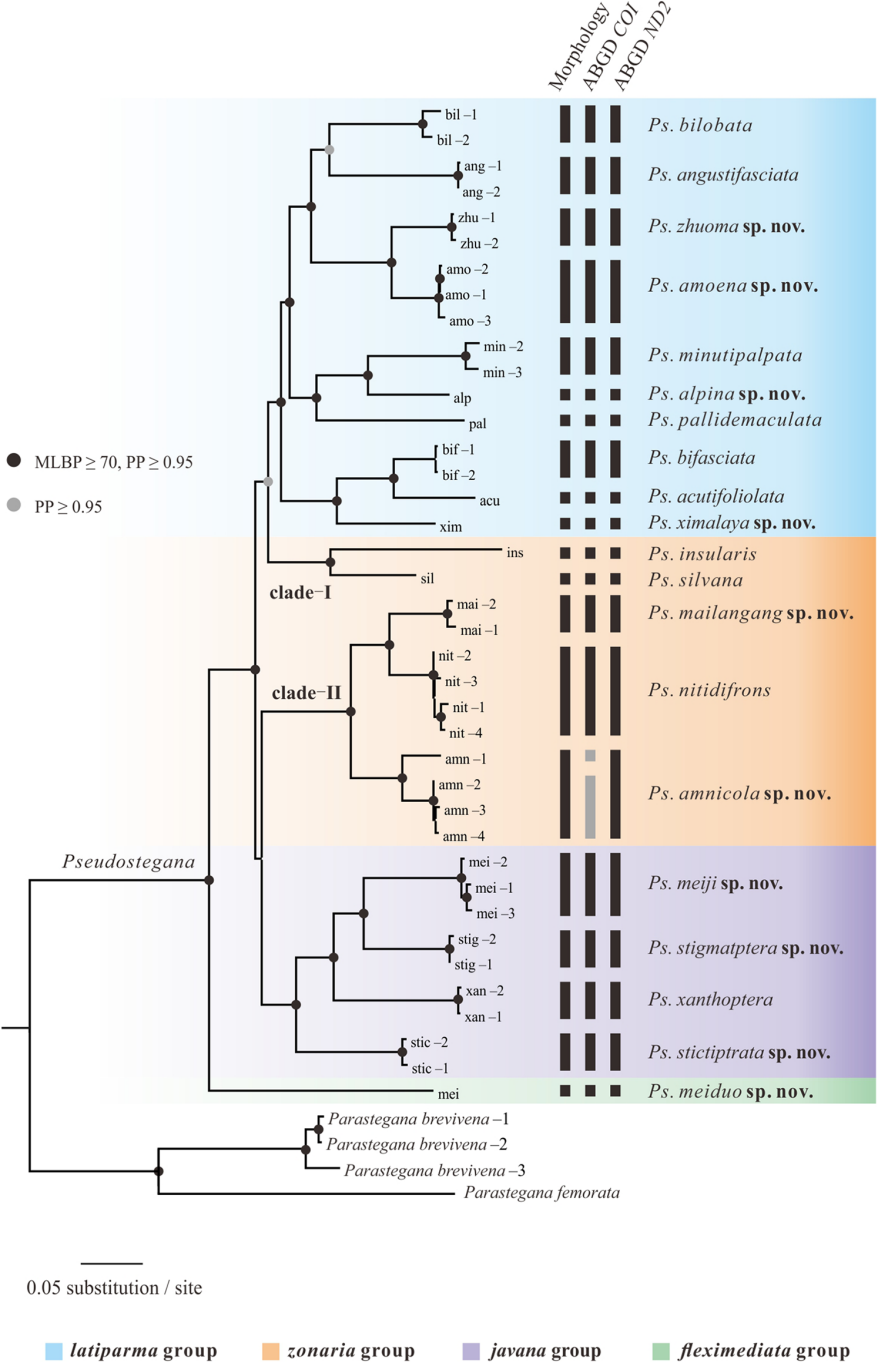
Maximum likelihood tree of the genus *Pseudostegana* inferred from the *COI*, *ND2* and *28S* sequences. The vertical bars represent the operational taxonomic units.

**Figure 3:**
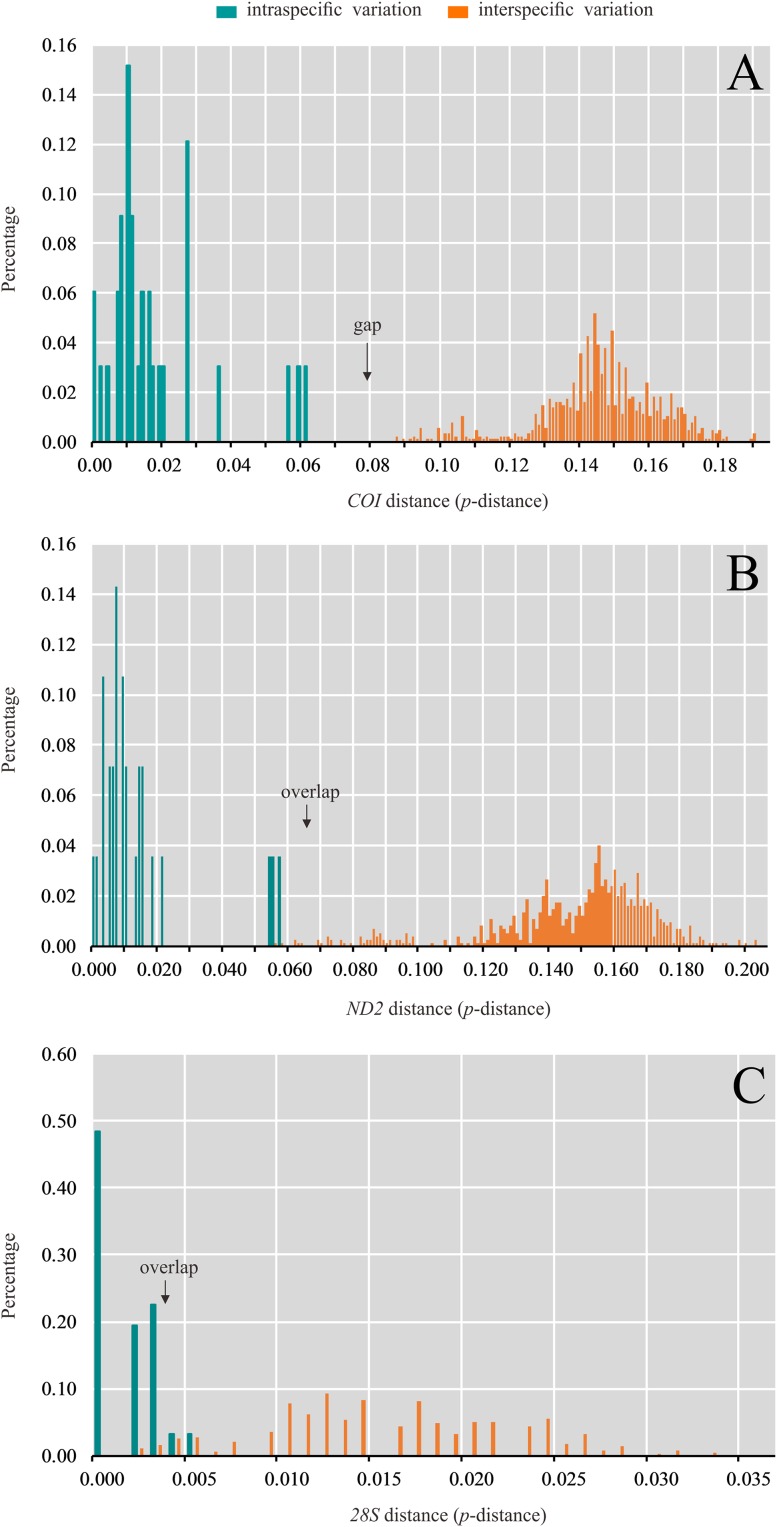
Distribution of intra- and interspecific pairwise genetic distance (*p*-distance) based on (A) *COI*, (B) *ND2* and (C) *28S* data set.

In general, the ML (Fig. 2) and Bayesian trees (Fig. S1) were similar in their topologies, especially at the terminal branches. Based on the phylogeny, *Pseudostegana* were recovered as a monophyletic genus with strong support. Within *Pseudostegana*, the monophyly of the *fleximediata*, *latiparma* and *javana* group all received strong support. The *zonaria* group was separated in two clades: (I) *Pseudostegana insularis* + *Pseudostegana silvana* and (II) *Pseudostegana nitidifrons* + *Pseudostegana amnicola* sp. nov. + *Pseudostegana mailangang* sp. nov. The monophyletic status of *zonaria* group is not supported by the Bayesian and ML analyses, as *Pseudostegana insularis* and *Pseudostegana silvana* were phylogenetically more closely related to the *latiparma* group (Fig. 3 and Fig. S1). At the species level, the phylogeny of the combined data set yielded 13 monophyletic clades and seven singletons with strong support.

### Species delimitation

The values of genetic variation (K2P distance) across taxonomic level are summarized (Table S3, S4 and S5). Intraspecific genetic variation calculated using *COI* ranged from 0.0% to 6.0% and the maximum intraspecific variation was detected in *P. amnicola*. In most cases, small intraspecific distances (<1%) were observed. The interspecific genetic variation ranged from 8.6% to 19.0% and the minimal interspecific genetic variation exceeded the maximum intraspecific genetic variation (Fig. 3A; Table S3). Intraspecific genetic variation calculated using *ND2* ranged from 0.0% to 5.6% and the maximum intraspecific variation was also detected in *P. amnicola*. The interspecific genetic variation ranged from 5.6% to 20.2%. Thus the intraspecific and interspecific genetic variation slightly overlapped (Fig. 3B; Table S4). Compared to the mitochondrial genes, genetic variation for the nuclear gene *28S* was small. The interspecific genetic variation ranged from 0.0% to 0.5%, while interspecific genetic variation ranged from 0.1% to 3.3%. The intraspecific and interspecific genetic variation largely overlapped (Fig. 3C; Table S5).

Species delimitation with the ABGD method based on the *COI* and *ND2* resulted in 22 (Table S6) and 20 (Table S7) molecular operational taxonomic units (MOTUs), respectively. These two mitochondrial fragment were largely congruent in most of the MOTUs, while the analysis based on *COI* fragment divided *P. amnicola* to two groups (Fig. 2). The analysis based on the *28S* data set yielded the lowest number of MOTUs (Table S8).

### Systematic accounts

Genus *Pseudostegana* Okada
*Stegana* (*Pseudostegana*) Okada, 1978: 392; Okada, 1982: 39. Type species: *Stegana* (*Parastegana*) *grandipalpis* Takada & Momma, 1975.
*Pseudostegana*: Sidorenko, 2002: 14 (as a genus); Chen, Toda & Wang, 2005: 407; Li, Gao & Chen, 2010: 1402.

*Diagnosis*: Arista with one ventral branch except for terminal fork; subvibrissa longer than half the length of vibrissa; first tarsomere of foreleg with five or six black, short and thick setae basally; aedeagus lacking outer membrane (modified from Chen, Toda & Wang, 2005).

**Figure 4:**
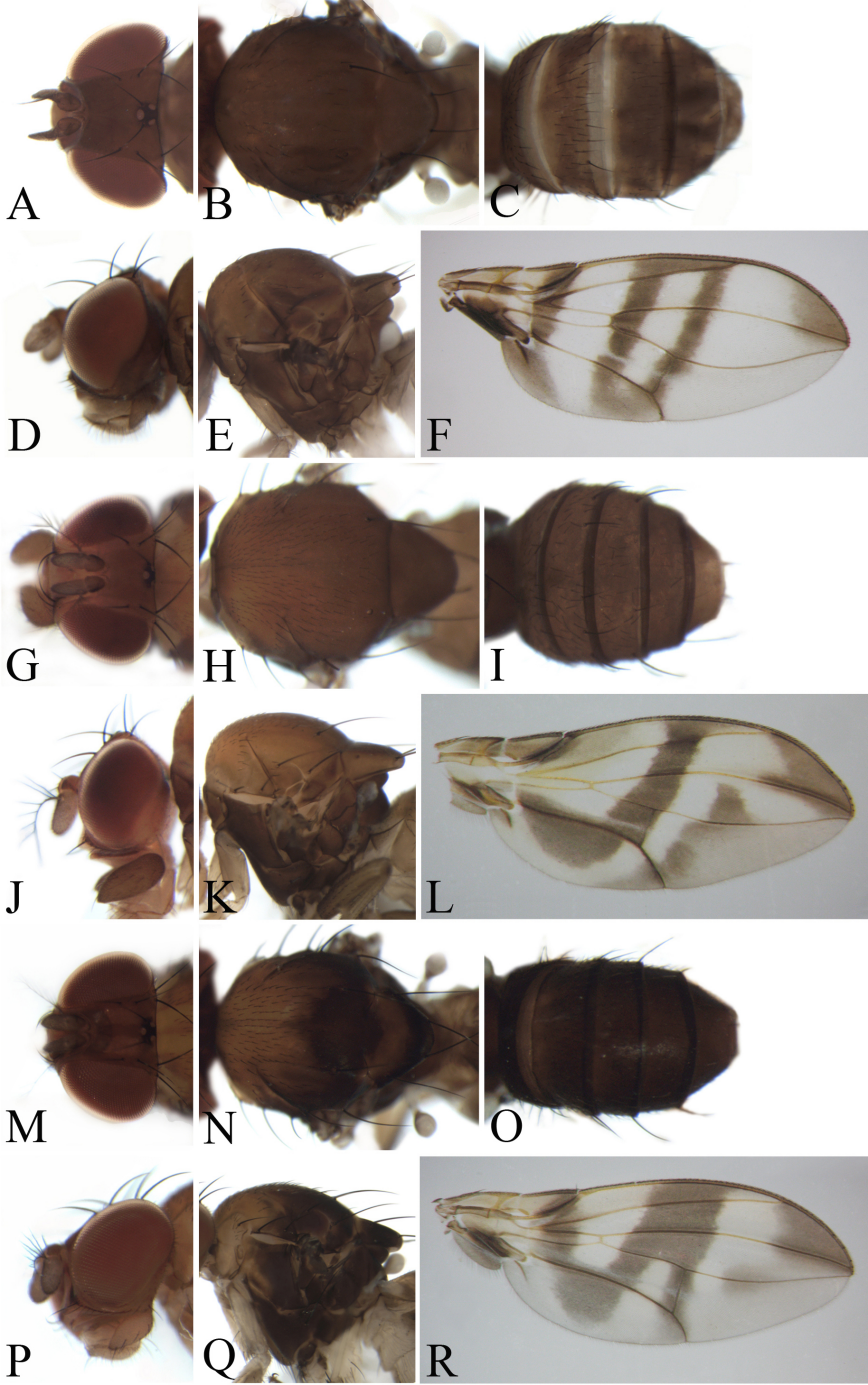
Morphological structures of female; frons, palpus, mesonotum, pleura, wing and abdominal tergites. (A–F) *Pseudostegana meiduo* sp. nov. Morphological structures of male frons, palpus, mesonotum, pleura, wing and abdominal tergites: (G–L) *Pseudostegana meiji* sp. nov.; (M–R) *Pseudostegana stictiptrata* sp. nov. Photo credit: Yuan Zhang.

**Figure 5:**
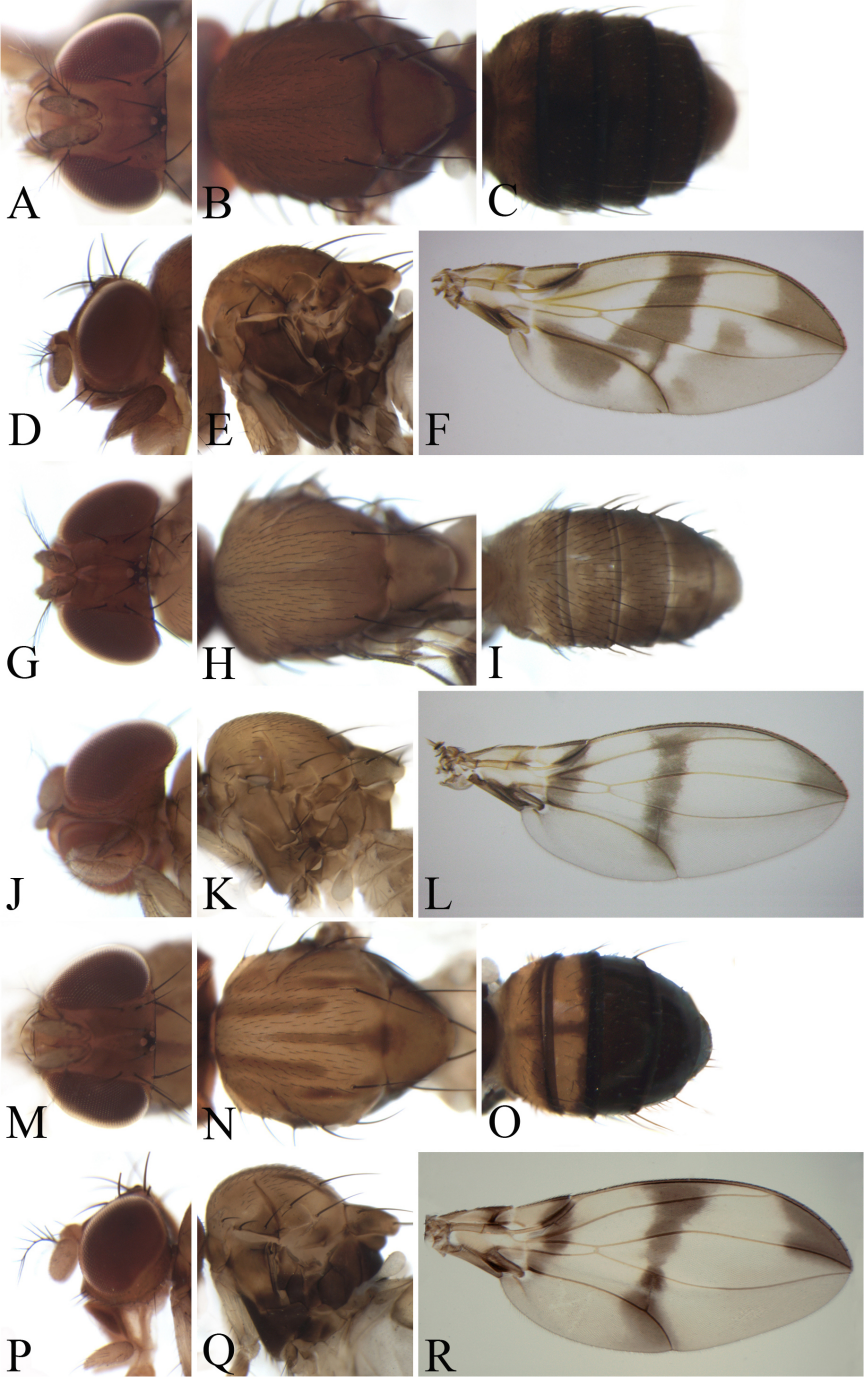
Morphological structures of male frons, palpus, mesonotum, pleura, wing and abdominal tergites. (A–F) *Pseudostegana stigmatptera* sp. nov.; (G–L) *Pseudostegana alpina* sp. nov.; (M–R) *Pseudostegana amoena* sp. nov. Photo credit: Yuan Zhang.

**Figure 6:**
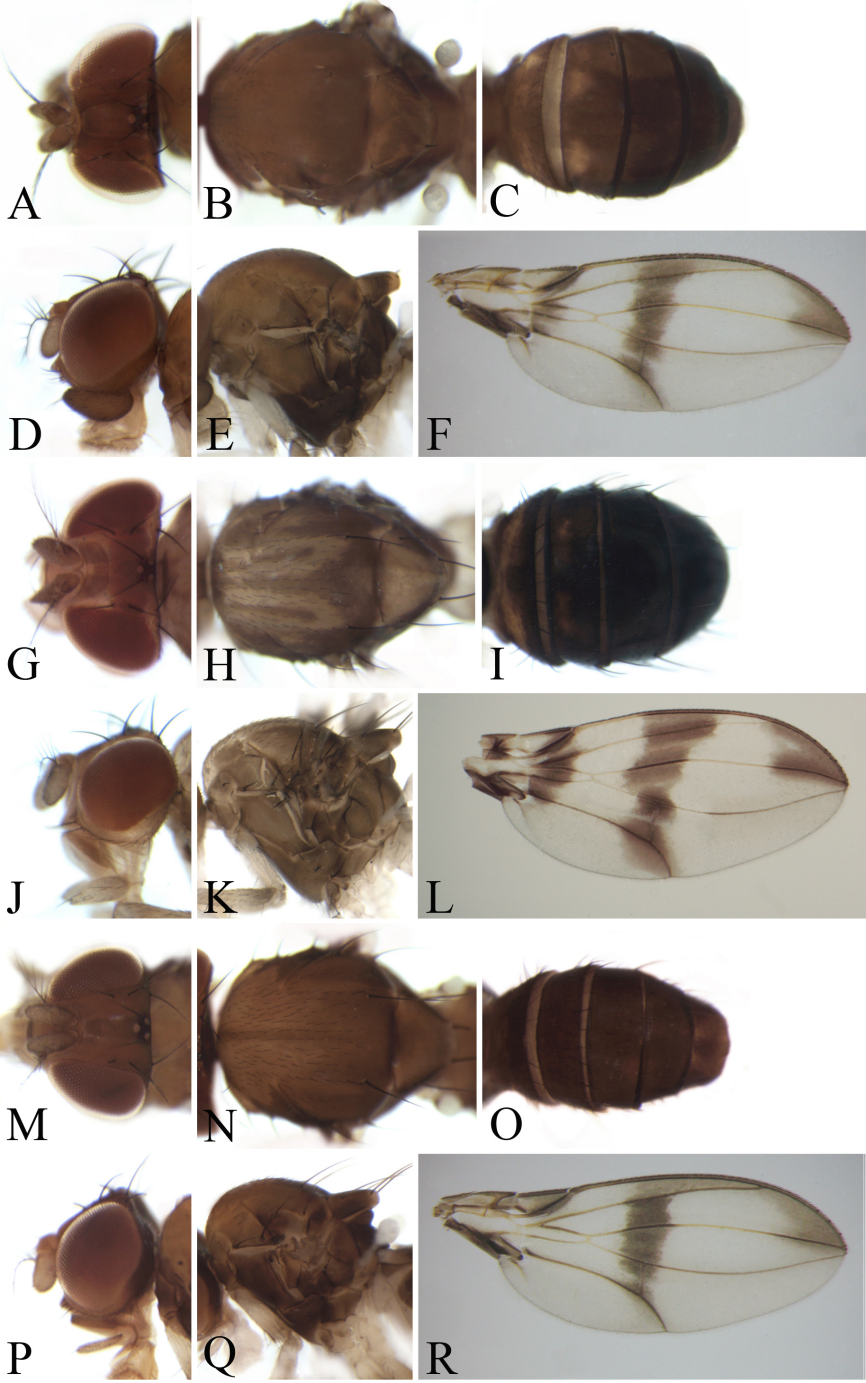
Morphological structures of male frons, palpus, mesonotum, pleura, wing and abdominal tergites. (A–F) *Pseudostegana ximalaya* sp. nov.; (G–L) *Pseudostegana zhuoma* sp. nov.; (M–R) *Pseudostegana amnicola* sp. nov. Photo credit: Yuan Zhang.

**Figure 7:**
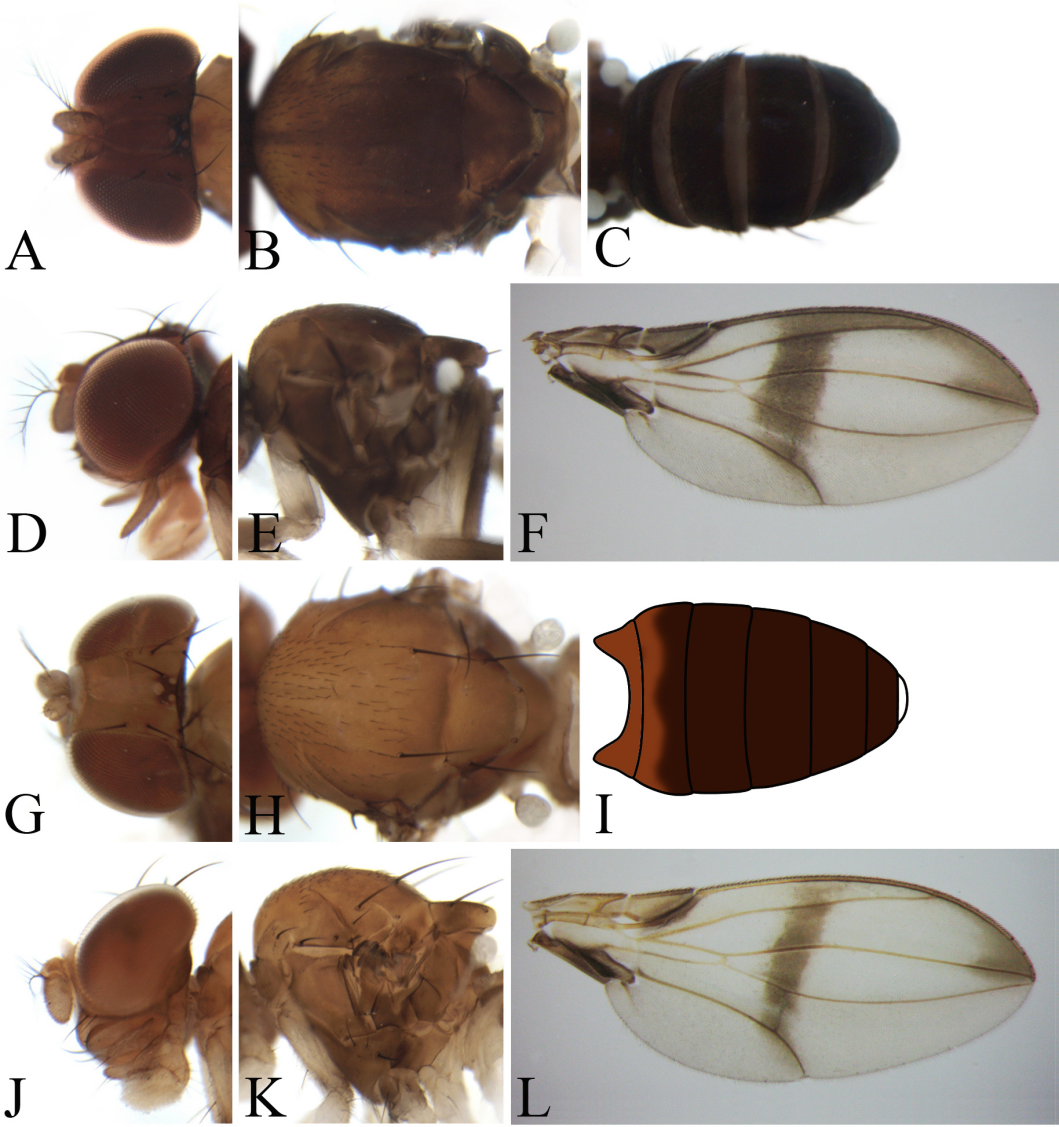
Morphological structures of male frons, palpus, mesonotum, pleura, wing and abdominal tergites. (A–F) *Pseudostegana mailangang* sp. nov.; (G–L) *Pseudostegana mystica* sp. nov. Photo credit: Yuan Zhang.

**Figure 8:**
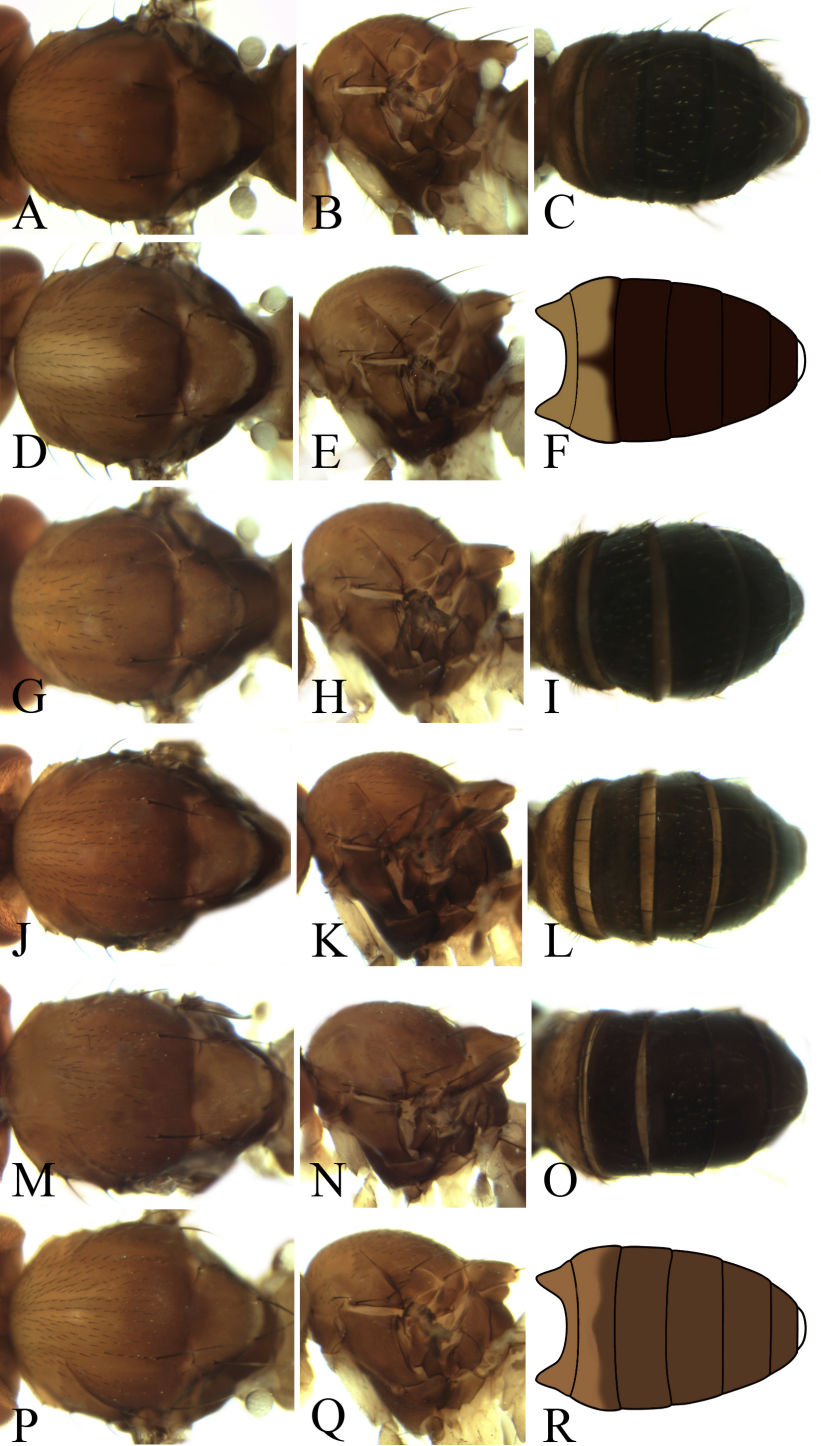
Morphological structures of male mesonotum, pleura and abdominal tergites. (A–C) *Pseudostegana acutifoliolata* Li, Gao & Chen, 2010; (D–F) *Pseudostegana angustifasciata* Chen & Wang, 2005; (G–I) *P. bifasciata* Chen & Wang, 2005; (J–L) *Pseudostegana bilobate* Li, Gao & Chen, 2010; (M–O). *Pseudostegana minutipalpata* Li, Gao & Chen, 2010; (P–R) *Pseudostegana pallidemaculata* Chen & Wang, 2005. Photo credit: Yuan Zhang.

**Figure 9:**
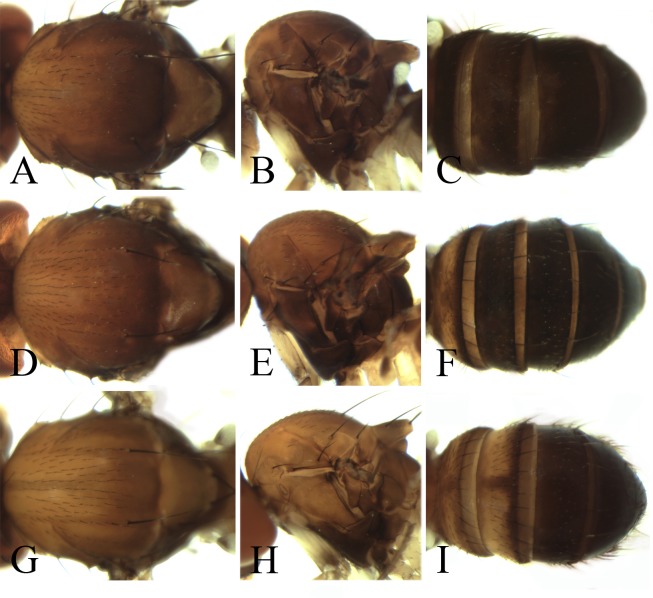
Morphological structures of male mesonotum, pleura and abdominal tergites. (A–C) *Pseudostegana insularis* Li, Gao & Chen, 2010; (D–F) *Pseudostegana nitidifrons* Chen & Wang, 2005; (G–I) *Pseudostegana silvana* Li, Gao & Chen, 2010. Photo credit: Yuan Zhang.

*Description*: Male and female (Figs. 4–9). **Head**: Eye brownish red. Ocellar triangle sometimes broadly or narrowly elongated to anterior margin of frons. Frons mostly glabrous, lacking minute, interfrontal setulae. Anterior reclinate orbital seta minute; posterior reclinate orbital seta situated nearer to proclinate seta than to inner vertical. Arista with long, dorsal branches. Clypeus brown to black. Subvibrissa mostly longer than half the length of vibrissa. Palpus slender in female, variable in male. **Thorax**: Mesonotum and scutellum dorsally convex. Katepisternal setae two or three; medial one shortest. Scutellum usually pale at tip; subscutellum swollen. **Wing**: Basal medial-cubital crossvein absent. Costal vein extending beyond tip of R4+5 vein, with five to seven peg-like spinules on ventral surface between veins R2+3 and R4+5. R2+3 vein slightly curved to costa at tip; M vein strongly convergent with R4+5 vein. Halter: stalk grayish; knob white. **Legs**: Mostly yellow, slender; mid tibia basally without strong, postero-dorsal setae. **Abdomen**: Sternites usually yellow to brown. **Male terminalia**: Epandrium broad, sometimes slightly constricted mid-dorsally, pubescent except for anterior margin. Surstylus separated from epandrium, mostly lacking pubescence, with several setae on outer and inner surfaces. Cercus separated from epandrium, pubescent and setigerous. Hypandrium broad, large, laterally mostly with one pair of paramedian setae, mid-anteriorly connected with apical part of aedeagal apodeme by aedeagal guide. Paramere with two long sensilla distally and several, small sensilla. Gonopods forming postero-median lobe, baso-laterally contiguous to parameres. Aedeagus usually with one pair of flap-like, serrated processes basally. Aedeagal apodeme long, rod-shaped, basally laterally flattened.

The *fleximediata* species group

*Diagnosis*: Ocellar triangle not elongated (Fig. 4A); M vein strongly curved after dm-cu crossvein (Fig. 4F); medial, dark color band much narrower than a half distance between r-m and dm-cu crossveins (Fig. 4F); seventh tergite with slender processes laterally in male.

***Pseudostegana meiduo* Zhang & Chen, sp. nov.**
urn:lsid:zoobank.org:act:CD5E03C7-3130-4FF6-9A1B-C4FE6690B7ED
(Figs. 4A–4F)

*Diagnosis*: This species obviously differs from the other *fleximediata* species by the wing having an annular patch medially (Fig. 4F), and distinct cross band subbasally (Fig. 4F).

*Description*: Female. **Head**: Ocellar triangle not elongated (Fig. 4A). Front brownish (Fig. 4A). Face grayish brown medially, dark brown laterally, black on lower corners. Clypeus and gena dark brown. Palpus brown, slightly broad (Fig. 4D). **Thorax**: Mesonotum and scutellum nearly brown (Fig. 4B). Pleura dark brown (Fig. 4E). **Legs**: Yellow, brown on forefemur and hind tibia, brown to dark brown on mid- and hindleg femora. **Abdomen**: All abdominal tergites nearly brown (Fig. 4C). Sternites yellow.

*Measurements*: BL = 2.40 mm in holotype, ThL = 1.25 mm, WL = 2.63 mm, WW = 1.10 mm, arb = 8/1, avd = 1.17, adf = 1.61, flw = 1.82, FW/HW = 0.43, ch/o = 0.09, prorb = 1.10, rcorb = 1.08, vb = 0.48, dcl = 0.29, sctl = 1.44, sterno = 0.70, orbito = 0.79, dcp = 0.13, sctlp = 1.11.

*Type specimen*: Holotype female (SCAU, no. 124842), CHINA: Beibeng, Motuo, Xizang, 29°19′N, 95°20′E, 1,000 m, 2.x.2010, *ex*. fallen logs, L Wu.

*Etymology*: The name means “flower” in Tibetan.

*Distribution*: China (Xizang).

The *javana* species group

*Diagnosis*: Wing with basal and medial cross bands fused posteriorly, forming V-shaped pattern (Figs. 4L, 4R and 5F).

**Figure 10:**
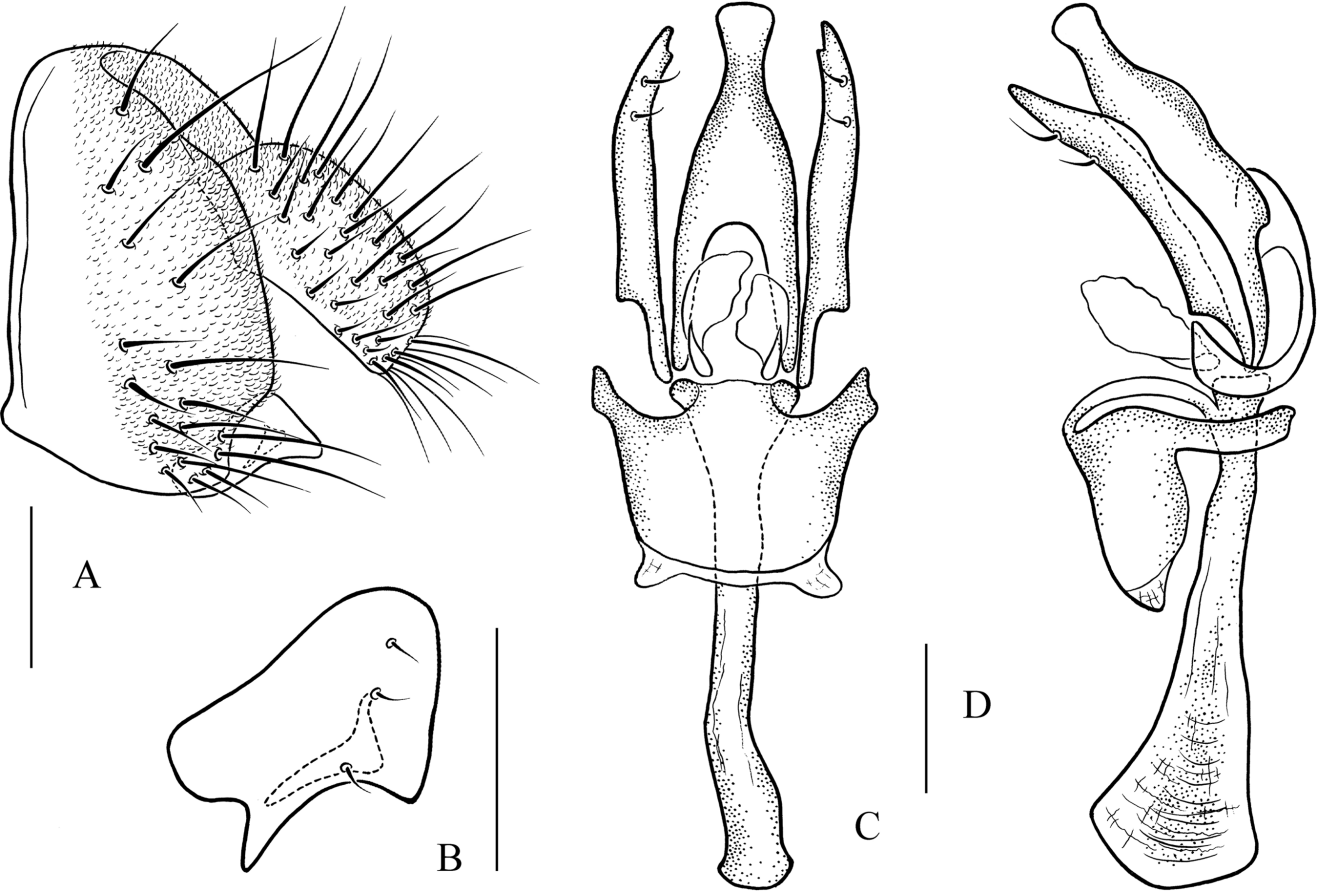
*Pseudostegana meiji* sp. nov., male terminalia. (A) Epandrium (epan), cercus (cerc) and surstylus (sur) (lateral view); (B) surstylus (ventral view); (C) hypandrium (hypd), paramere (pm), gonopods (gon), aedeagus (aed) and aedeagal apodeme (aed a) (ventral view); (D) ditto (lateral view). Scale bars = 0.1 mm. Drawing credit: Yuan Zhang.

***Pseudostegana meiji* Zhang & Chen, sp. nov.**
urn:lsid:zoobank.org:act:0E0F9E07-C2E7-48F6-B021-F699E21F88CC
(Figs. 4G–4L and 10)

*Diagnosis*: This species is similar to *Pseudostegana stigmatptera* sp. nov. by the wing patches (Fig. 4L) and male terminalia (Fig. 10), but can be distinguished by the surstylus: in this species, the surstylus is strongly protruded on posterior corner in lateral view (Fig. 10A), broadened, and approximately one and half times as high as wide (Fig. 10B); compare with *Pseudostegana stigmatptera* sp. nov. The 5.8% *COI* interspecific genetic distance to *P. yiqini* is one of the smallest interspecific distances ascertained within this subgenus.

*Description*: Male and female. **Head**: Ocellar triangle not elongated (Fig. 4G). Frons brown in male (Fig. 4G), yellowish brown in female. Face and gena yellow. Clypeus yellow medially, black laterally. Palpus brown in both sexes, broad, large in male, medially about one-third as wide as long, slender in female (Fig. 4J). **Thorax**: Mesonotum yellow in male (Fig. 4H), brownish yellow in female; scutellum brown (Fig. 4H). Pleura dark brown in male (Fig. 4K), dark brown to black in female. **Legs**: Mostly yellow; brown to dark on mid- and hindleg femora. **Abdomen**: All tergites brown (male) to dark brown (female), yellow latterly (Fig. 4I). Sternites brownish yellow in male, brown in female. **Male terminalia**: Epandrium with ∼18 setae (Fig. 10A). Hypandrial paramedian setae absent (Fig. 10C). Paramere shallowly bifurcated apically (Fig. 10C). Aedeagus round apically, with membranous processes basally (Figs. 10C and 10D).

*Measurements*: BL = 2.85 mm in holotype (range in five males and five females paratypes, 2.63–3.01 mm in males, 2.53–2.93 mm in females), ThL = 1.25 mm (1.26–1.43 mm in males, 1.00–1.46 mm in females), WL = 2.43 mm (2.47–2.67 mm in males, 2.07–2.63 mm in females), WW = 1.06 mm (1.10–1.20 mm in males, 1.00–1.20 mm in females), arb = 7/1 (6–8/1), avd = 0.72 (0.86–1.00), adf = 2.63 (1.83–2.24), flw = 2.37 (1.70–2.07), FW/HW = 0.39 (0.39–0.55), ch/o = 0.09 (0.06–0.09), prorb = 0.85 (0.79–0.96), rcorb = 0.93 (0.86–1.21), vb = 0.73 (0.64–0.95), dcl = 0.30 (0.31–0.35), sctl = 1.13 (0.89–1.05), sterno = 0.48 (0.60–1.00), orbito = 0.98 (1.00–1.03), dcp = 0.17 (0.18–0.16), sctlp = 1.24 (0.94–1.21).

*Type specimens*: Holotype male (SCAU, no. 122031), CHINA: Muyiji Park, Ximeng, Yunnan, 22°37′N, 99°36′E, 1,100 m, 2.iv.2011, *ex.* fallen logs, YR Su. Paratypes: CHINA: two females (SCAU, nos. 122032, 33), same data as holotype; 10 males, 15 females (five males and five females in KIZ, nos. 0088165–74; the rest in SCAU, nos. 124844–58), Muyiji, Ximeng, Yunnan, 22°37′N, 99°36′E, 1,200 m, 29.iv. −3.v.2016, *ex*. tussock, J Huang, YQ Liu, YL Wang, L Zhu; eight males, 14 females (SCAU, nos. 124859–80), Mengdong, Cangyuan, Yunnan, 23°10′N, 99°14′E, 1,320 m, 7.v.2016, *ex*. tussock, J Huang, YQ Liu, YL Wang, L Zhu; one male, (SCAU, no. 124881), Botanic Garden, Ruili, Yunnan, 24°1′N, 97°51′E, 1,174 m, 22.viii.2016, *ex*. tussock, L Gong; one male, two females (SCAU, nos. 111353–55), Santaishan, Mangshi, Yunnan, 24°19′N, 98°20′E, 900 m, 4.xi.2017, *ex*. tussock, HW Chen, L Gong, BX Li; one male, one female (SCAU, nos. 111356, 57), Moli Forest Park, Ruili, Yunnan, 24°7′N, 97°59′E, 920 m, 5.xi.2017, L Gong, BX Li.

*Etymology*: The name means “great deity” in the language of the Wa nationality in Yunnan Province.

*Distribution*: China (Yunnan).

**Figure 11:**
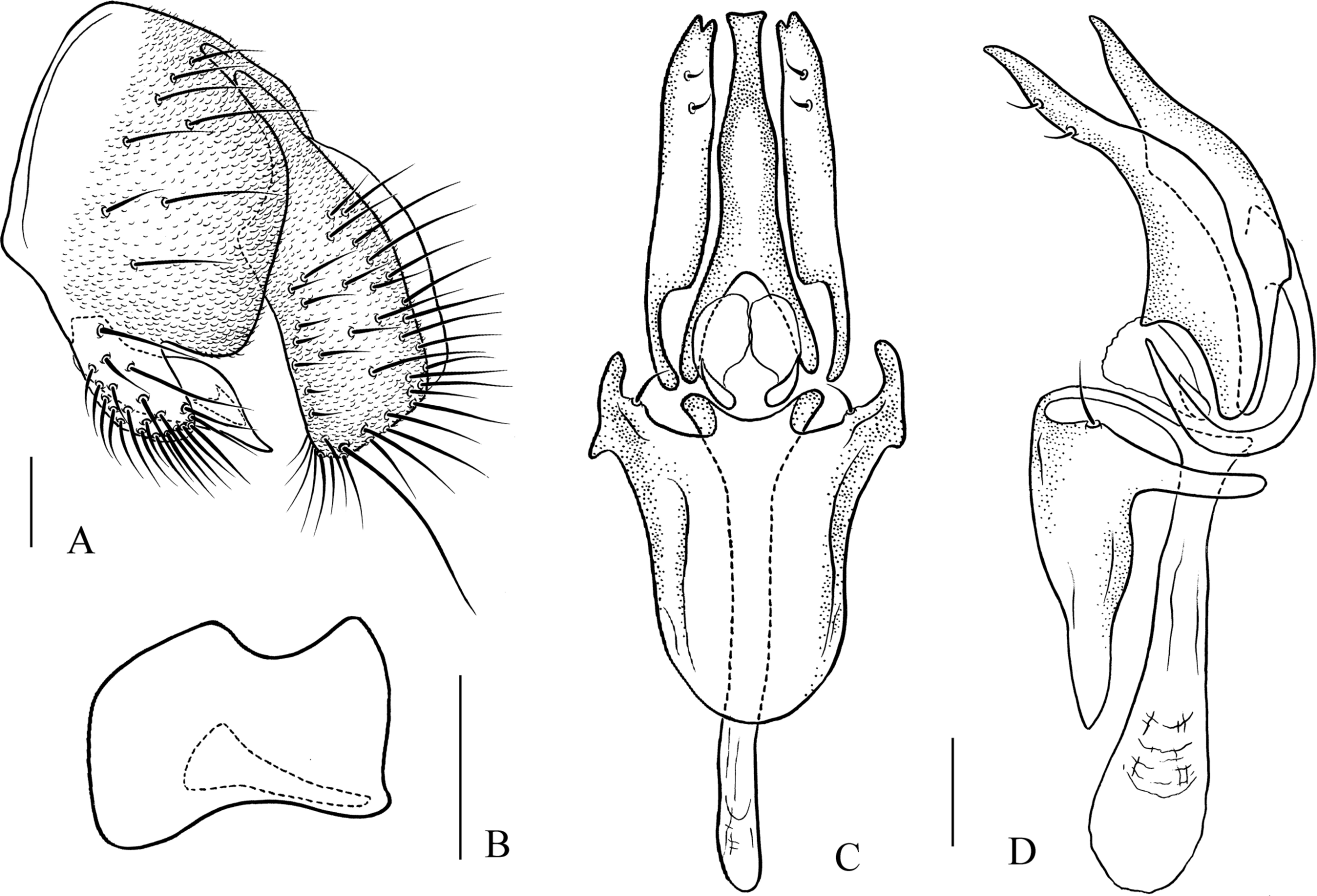
*Pseudostegana stictiptrata* sp. nov., male terminalia. (A) Epandrium, cercus and surstylus; (B) surstylus (ventral view); (C) hypandrium, paramere, gonopods, aedeagus and aedeagal apodeme (ventral view); (D) ditto (lateral view). Scale bars = 0.1 mm. Drawing credit: Yuan Zhang.

***Pseudostegana stictiptrata* Zhang & Chen, sp. nov.**
urn:lsid:zoobank.org:act:88C101ED-F994-4E0A-8CCB-B2F60378412F
(Figs. 4M–4R and 11)

*Diagnosis*: This species differs from the other *javana* species by the wing pattern (Fig. 4R), and the epandrium being roundly protruded ventrally, with dense setae (Fig. 11A).

*Description*: Male. **Head**: Ocellar triangle elongated (Fig. 4M). Frons yellow to yellowish brown (Fig. 4M). Face brown. Clypeus brown medially, black laterally. Gena orange brown. Palpus yellow, broad, large in male, medially about one-third as wide as long (Fig. 4P). **Thorax**: Mesonotum orange–yellow on anterior one-third to half, black on posterior half to one-third (Fig. 4N); scutellum and pleura black (Fig. 4N). Pleura dark brown to black (Fig. 4Q). **Legs**: Mostly yellow, dark brown distally on all femora. **Abdomen**: All tergites dark brown to black (Fig. 4O). Sternites yellow on second and third, brownish fourth to sixth. **Male terminalia**: Epandrium with numerous setae (Fig. 11A). Cercus slightly protruded ventrally (Fig. 11A). Surstylus strongly protruded on posterior corner in lateral view (Fig. 11A), broadened, approximately one and a half times as high as wide (Fig. 11B). Hypandrium with one pair of paramedian setae (Figs. 11C and 11D). Paramere shallowly bifurcated apically (Figs. 11C and 11D). Aedeagus apically slightly concave in ventral view, with membranous processes basally (Figs. 11C and 11D).

*Measurements*: BL = 3.39 mm in holotype (range in four males paratypes: 2.40–2.83 mm), ThL = 1.52 mm (1.25–1.39 mm), WL = 3.03 mm (2.57–2.89 mm), WW = 1.36 mm (1.12–1.28 mm), arb = 8/1 (6–8/1), avd = 0.69 (0.81–0.87), adf = 2.08 (1.83–2.33), flw = 1.86 (1.52–2.00), FW/HW = 0.46 (0.35–0.42), ch/o = 0.08 (0.06–0.10), prorb = 0.78 (0.84–1.04), rcorb = 0.94 (0.80–0.87), vb = 0.56 (0.80–1.08), dcl = 0.29 (0.25–0.33), sctl = 0.94 (0.98–1.04), sterno = 0.71 (0.79–0.87), orbito = 1.20 (0.96–1.11), dcp = 0.17 (0.22), sctlp = 1.28 (1.11–1.25).

*Type specimens*: Holotype male (SCAU, no. 122034), CHINA: Hesong, Menghai, Yunnan, 21°49′N, 100°06′E, 1,700 m, 12.v.2012, *ex*. stone with moss, HW Chen. Paratypes: CHINA: two males (SCAU, nos. 122035, 36), same data as holotype; two males (SCAU, nos. 124882, 83), Yakou, Huanglianshan, Lvchun, Yunnan, 22°50′N, 102°17′E, 1,900 m, 31.x.2016, *ex.* tussock, HW Chen; one male (KIZ, 0090510), Daweishan, Pingbian, Yunnan, 22°92′N, 103°68′E, 1,700–1,900 m, 12.viii.2017, *ex*. tussock, HW Chen.

*Etymology*: A combination of the Greek words: “stict” + “pteron,” referring to the wing having pattern.

*Distribution*: China (Yunnan).

**Figure 12:**
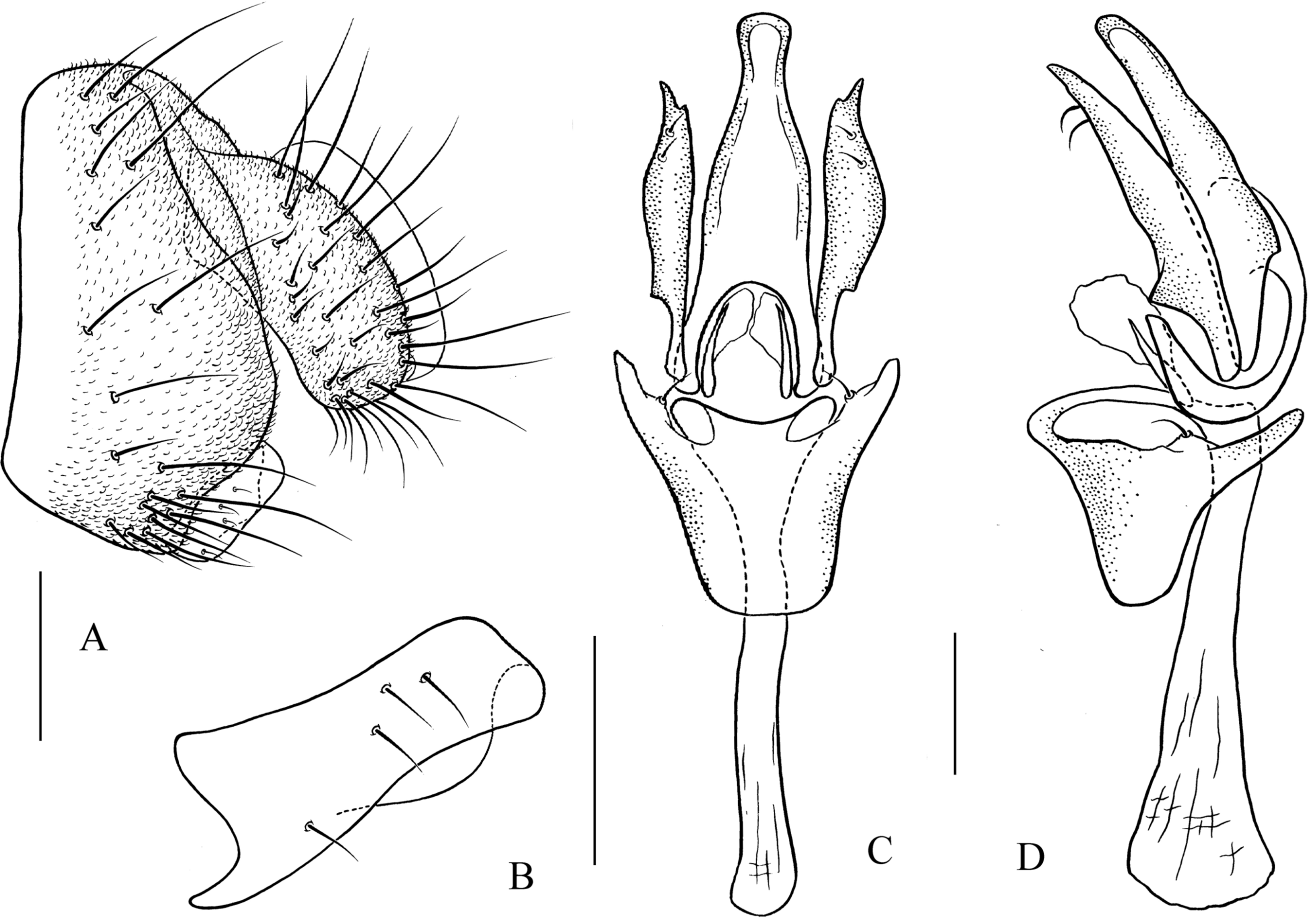
*Pseudostegana stigmatprata* sp. nov., male terminalia. (A) Epandrium, cercus and surstylus; (B) surstylus; (C) hypandrium, paramere, gonopods and aedeagus (ventral view); (D) ditto (lateral view). Scale bars = 0.1 mm. Drawing credit: Yuan Zhang.

***Pseudostegana stigmatptera* Zhang & Chen, sp. nov.**
urn:lsid:zoobank.org:act:72488FCE-4052-403B-A450-3A9312B4BB2F
(Figs. 5A–5F and 12)

*Diagnosis*: Surstylus strongly protruded and pointed on anterior corner, broadened, approximately two times as high as wide (Fig. 12B).

*Description*: Male and female. **Head**: Ocellar triangle not elongated (Fig. 5A). Frons brown (Fig. 5A). Face and gena yellow. Clypeus dark brown. Palpus brown yellow, broad, large in male, medially one-third as wide as long (Fig. 5D). **Thorax**: Mesonotum yellowish brown; scutellum brown, yellow at tip (Fig. 5B). Pleura dark brown (Fig. 5E). **Legs**: Mostly yellow, brown on all femora. **Abdomen**: All tergites black, sometimes laterally yellow on second to fourth (Fig. 5C). Sternites brown in male, dark brown in female. **Male terminalia**: Epandrium with ∼19 setae (Fig. 12A). Hypandrial paramedian setae present (Figs. 12C and 12D). Paramere shallowly bifurcated apically (Figs. 12C and 12D). Aedeagus apically round, with membranous processes basally (Figs. 12C and 12D).

*Measurements*: BL = 2.97 mm in holotype (range in five males and five females paratypes, 2.41–2.97 mm in males, 2.88–3.23 mm in females), ThL = 1.37 mm (1.15–1.42 in males, 1.42–1.50 mm in females), WL = 2.80 mm (2.41–2.79 mm in males, 2.73–2.89 mm in females), WW = 1.17 mm (1.13–1.30 mm in males, 1.23–1.35 mm in females), arb = 7/1 (6–7/1), avd = 0.97 (0.72–1.09), adf = 1.61 (1.67–1.96), flw = 2.03 (1.46–1.97), FW/HW = 0.47 (0.46–0.52), ch/o = 0.04 (0.06–0.09), prorb = 0.77 (0.74–0.91), rcorb = 0.96 (0.82–1.06), vb = 0.71 (0.73–0.89), dcl = 0.29 (0.21–0.30), sctl = 0.84 (1.02–1.11), sterno = 0.41 (0.43–0.71), orbito = 1.05 (0.87–1.11), dcp = 0.18 (0.16–0.26), sctlp = 1.05 (0.95–1.22).

*Type specimens*: Holotype male (SCAU, no. 122037), CHINA: Hesong, Menghai, Yunnan, 1,700–1,900 m, 16.iv.2010, *ex.* tussock, JJ Gao. Paratypes: CHINA: 25 males, 28 females (five males, five females in KIZ, nos. 0088175–82, 0090501–02; 20 males, 23 females in SCAU, nos. 122038–122080), *ex.* tree trunks, tussock and stone with moss, JJ Gao, YR Su, L Wang, L Wu, the rest same data as holotype; 26 males, 24 females (SCAU, nos. 122081–122130), 26.iii.2011, 7.iv.2011, 11.v.2012, *ex.* tree trunks, tussock and stone with moss, HW Chen, JM Lu, ZF Shao, YR Su, SJ Yan, same data as holotype; three males, three females (SCAU, nos. 122131–36), Baihualing, Baoshan, Yunnan, 25°18′N, 98°48′E, 1,400 m, 22.vi.2013, 24.viii.2013, *ex.* tree tussock, KY An, QS Gao, K Liu, JJ Liu.

*Etymology*: A combination of the Greek words: “stigma” + “pteron” meaning spotted wing.

*Distribution*: China (Yunnan).

The *latiparma* species group

*Diagnosis*: Wing subbasally with distinct cross band (Figs. 5L, 5R, 6F and 6L).

**Figure 13:**
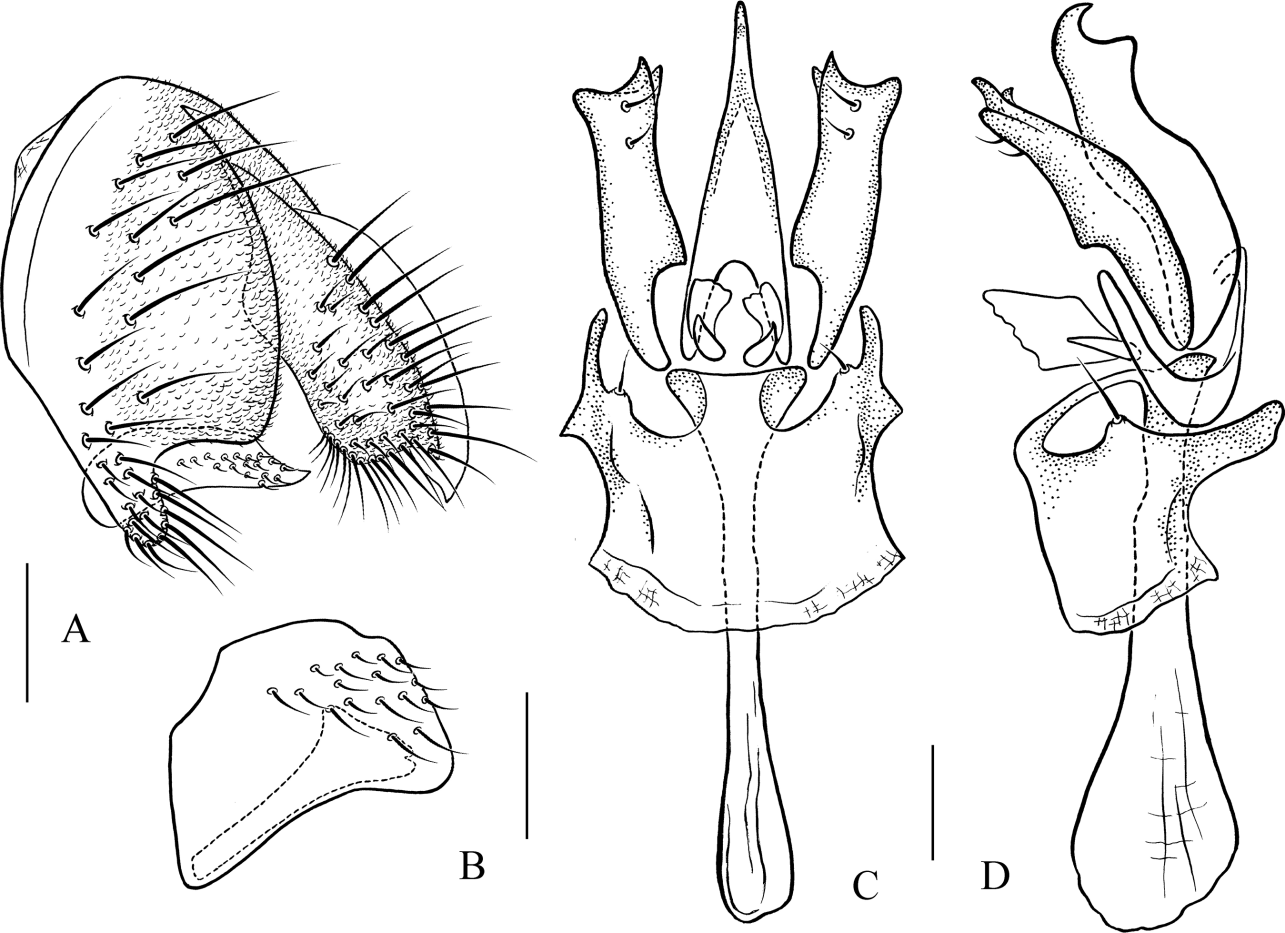
*Pseudostegana alpina* sp. nov., male terminalia. (A) Epandrium, cercus and surstylus; (B) surstylus; (C) hypandrium, paramere, gonopods, aedeagus and aedeagal apodeme (ventral view); (D) ditto (lateral view). Scale bars = 0.1 mm. Drawing credit: Yuan Zhang.

***Pseudostegana alpina* Zhang & Chen, sp. nov.**
urn:lsid:zoobank.org:act:31D32207-72FD-41A4-8971-8349404EB97C
(Figs. 5G–5L and 13)

*Diagnosis*: This species is related to *Pseudostegana minutipalpata* Li, Gao & Chen, 2010 from Yunnan by the wing pattern (Fig. 5L), and periphallic organs (Fig. 13A), but can be distinguished by the surstylus and aedeagus; in this species, the surstylus is protruded on anterior corner (Fig. 13B); aedeagus apically acute and subapically so strongly, dorsally protruded in lateral view (Fig. 13D).

*Description*: Male. **Head**: Ocellar triangle not elongated (Fig. 5G). Frons brown (Fig. 5G). Face and gena brown. Clypeus brown medially, black laterally. Palpus brown, slender (Fig. 5J). **Thorax**: Mesonotum medially yellow and with a thin brownish yellow, longitudinal stripe, laterally brownish yellow; scutellum brownish yellow (Fig. 5H). Pleura slightly glossy, brownish yellow above, dark brown below (Fig. 5K). **Legs**: Yellow, dark brown on all knees. **Abdomen**: All tergites brown, yellow on first to third medially (Fig. 5I). Sternites yellow. **Male terminalia**: Epandrium roundly protruded on ventral margin, with numerous setae (Fig. 13A). Surstylus slightly protruded on posterior corner (Fig. 13B). Cercus ventrally elongated in lateral view (Fig. 13A). Hypandrium with one pair of paramedian setae (Figs. 13C and 13D). Paramere acute, with one small projection apically, subapically broadened (Figs. 13C and 13D) Aedeagus apically slightly pointed in ventral view (Fig. 13C), basally with nearly membranous processes (Figs. 13C and 13D).

*Measurements*: BL = 2.89 mm in holotype, ThL = 1.24 mm, WL = 2.68 mm, WW = 1.23 mm, arb = 7/1, avd = 0.97, adf = 1.84, flw = 1.64, FW/HW = 0.41, ch/o = 0.08, prorb = 1.15, rcorb = 1.22, vb = 0.95, dcl = damage, sctl = 1.25, sterno = 0.69, orbito = 1.11, dcp = damage, sctlp = 1.05.

*Type specimen*: Holotype male (SCAU, no. 122158), CHINA: Hesong, Menghai, Yunnan, 1,800 m, 12.v.2012, *ex.* stone with moss, HW Chen.

*Etymology*: From the Latin word: alpinus, means high mountain, referring to the type locality.

*Distribution*: China (Yunnan).

**Figure 14:**
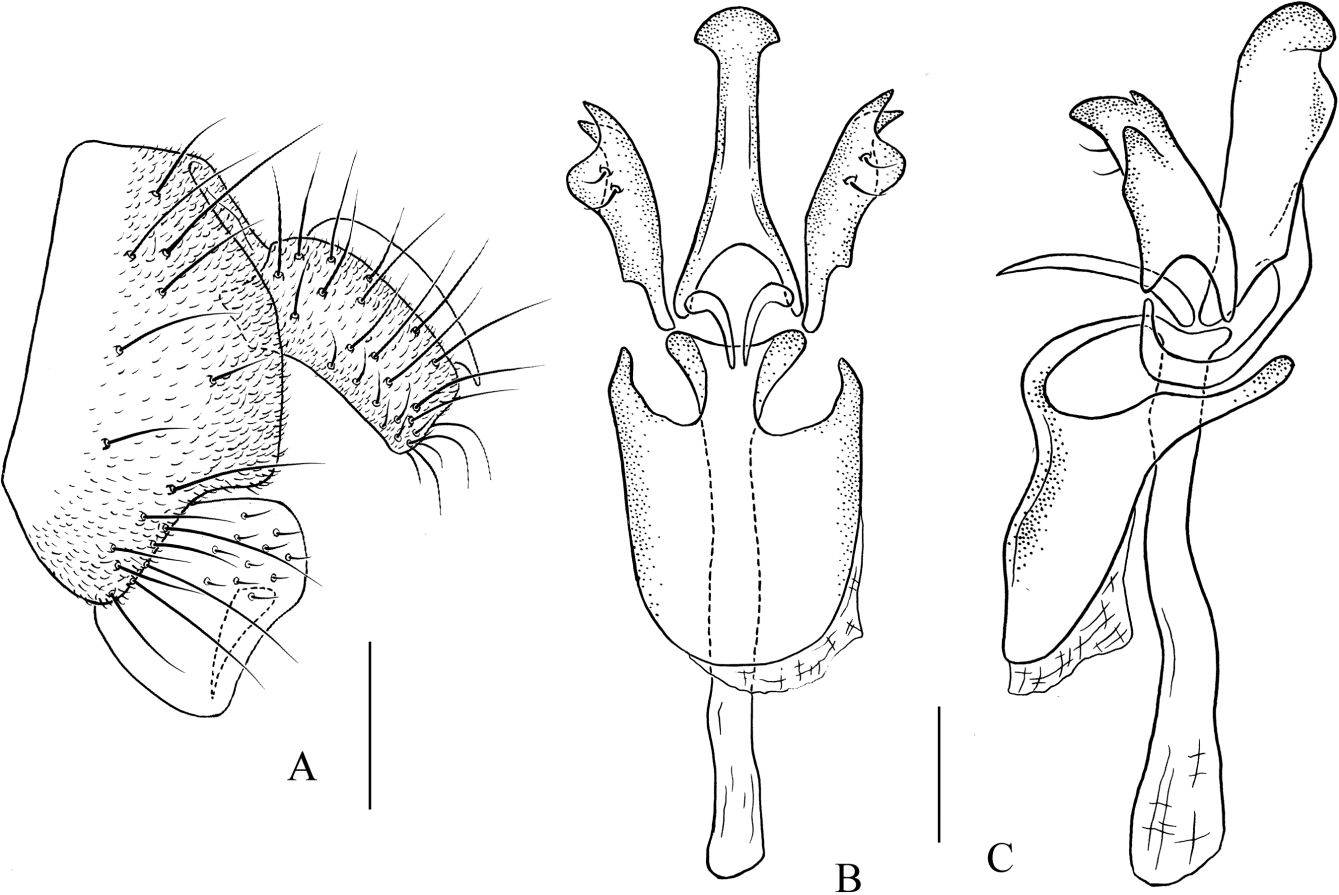
*Pseudostegana amoena* sp. nov., male terminalia. (A) Epandrium, cercus and surstylus; (B) hypandrium, paramere, gonopods, aedeagus and aedeagal apodeme (ventral view); (C) ditto (lateral view). Scale bars = 0.1 mm. Drawing credit: Yuan Zhang.

***Pseudostegana amoena* Zhang & Chen, sp. nov.**
urn:lsid:zoobank.org:act:D3696645-FA69-4B4B-A5D8-D6FCF6F90764
(Figs. 5M–5R and 14)

*Diagnosis*: This species is related to *Pseudostegana zhuoma* sp. nov. by the wing pattern (Figs. 5R and 6L) and male terminalia (Figs. 14 and 16); they can be distinguished by the yellow mesonotum, with five brown, longitudinal stripes (Fig. 5N); scutellum yellowish brown, yellow at tip (Fig. 5N); pleura brownish yellow anteriorly, black posteriorly (Fig. 5Q); aedeagus slightly smooth ventrally (Fig. 14C); compare with *Pseudostegana zhuoma* sp. nov.

**Figure 15:**
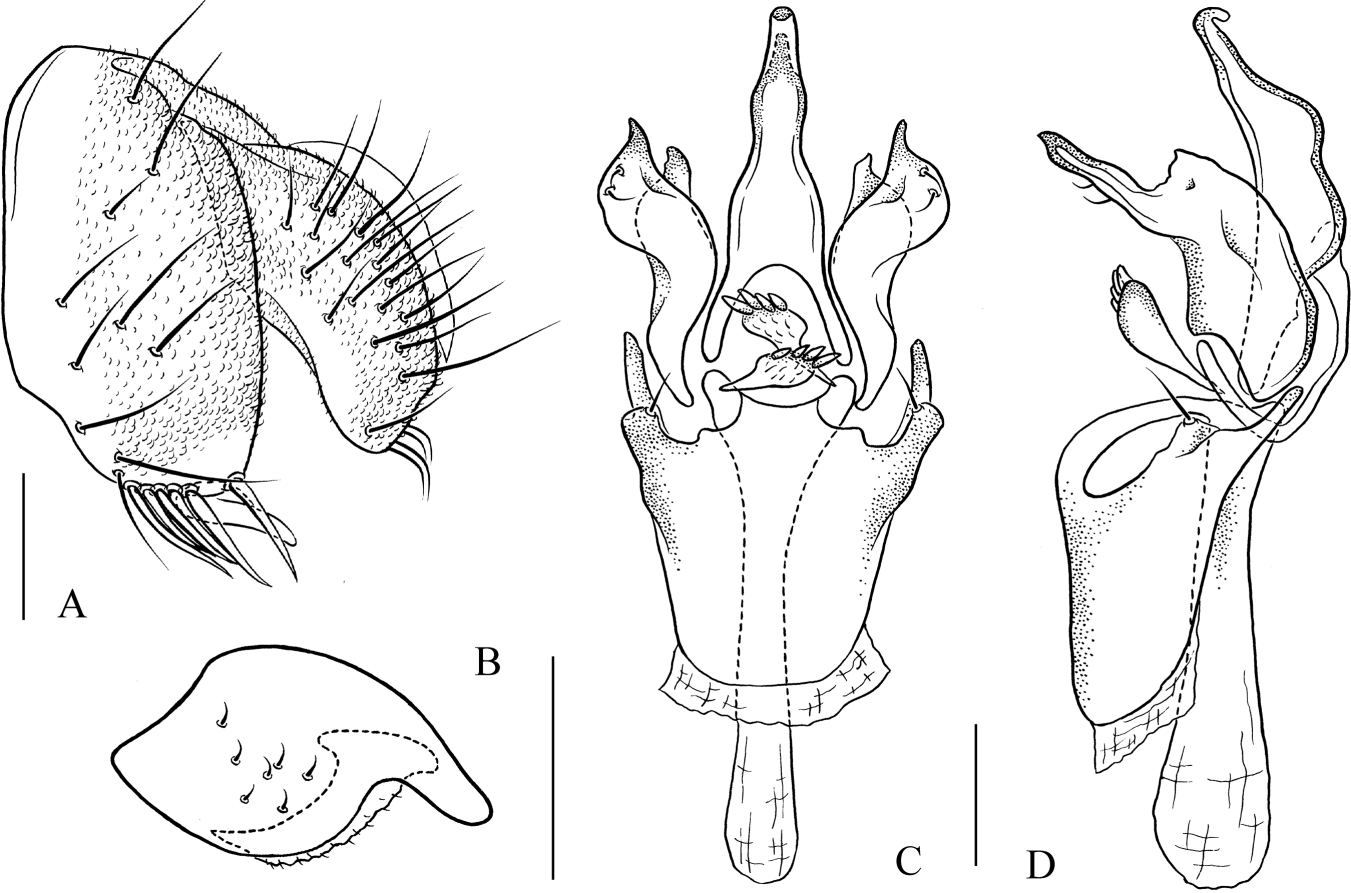
*Pseudostegana ximalaya* sp. nov., male terminalia. (A) Epandrium, cercus and surstylus; (B) surstylus; (C) hypandrium, paramere, gonopods, aedeagus and aedeagal apodeme (ventral view); (D) ditto (lateral view). Scale bars = 0.1 mm. Drawing credit: Yuan Zhang.

**Figure 16:**
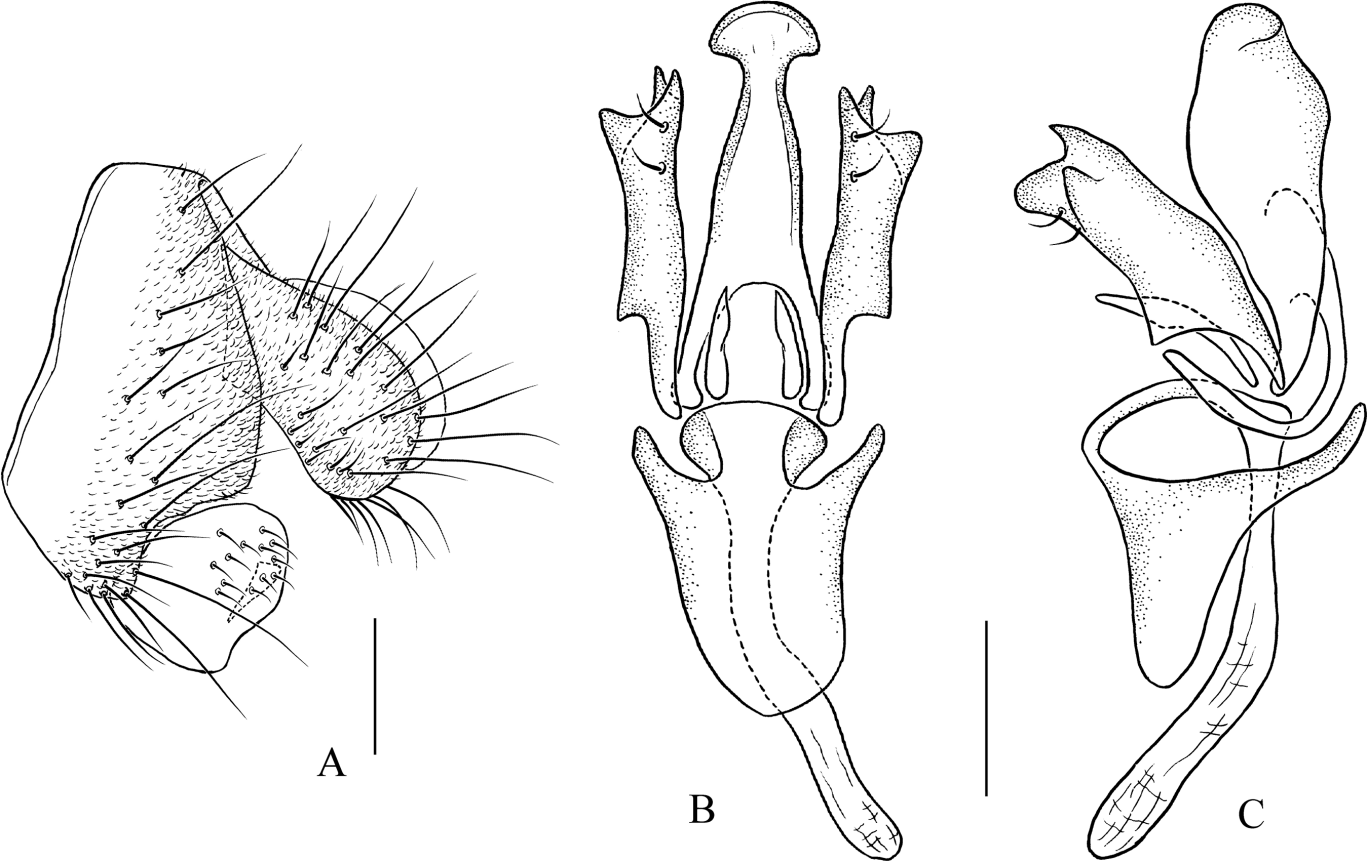
*Pseudostegana zhuoma* sp. nov., male terminalia. (A) Epandrium, cercus and surstylus; (B) hypandrium, paramere, gonopods, aedeagus and aedeagal apodeme (ventral view); (C) ditto (lateral view). Scale bars = 0.1 mm. Drawing credit: Yuan Zhang.

*Description*: Male and female. **Head**: Ocellar triangle narrowly elongated, yellow (Fig. 5M). Frons brown (Fig. 5M). Face yellowish brown. Clypeus brown to dark brown. Gena brown. Palpus brownish yellow to brown, slightly broad in male, medially a quarter as wide as long (Fig. 5P), yellow and slender female. **Legs**: Yellow. **Abdomen**: Tergites glossy, yellow on second and third tergites, black along lateral and posterior margins, with one brownish longitudinal stripe medially, the rest black (Fig. 5O). Sternites brown to dark brown. **Male terminalia**: Epandrium broadened on posterior margin, roundly protruded on ventral margin (Fig. 14A). Surstylus broadened, with several setae on outer surfaces (Fig. 14A). Hypandrial paramedian setae absent (Figs. 14B and 14C). Paramere round apically, brodened subapically, with one acute, dorsal projection apically (Figs. 14B and 14C). Aedeagus broadways expanded apically, slightly dorsad protruded submedially in lateral view, with rod-like processes basally (Figs. 14B and 14C).

*Measurements*: BL = 3.05 mm in holotype (range in five males and five females paratypes: 2.41–2.95 mm in males, 2.97–3.43 mm in females), ThL = 1.47 mm (0.96–1.25 mm in males, 1.25–1.51 mm in females), WL = 3.13 mm (2.30–2.93 mm in males, 2.97–3.07 mm in females), WW = 1.43 mm (1.03–1.37 mm in males, 1.33–1.45 mm in females), arb = 6/1 (5–7/1), avd = 1.60 (0.85–1.67), adf = 1.73 (1.47–1.92), flw = 0.93 (0.99–2.09), FW/HW = 0.47 (0.36–0.48), ch/o = 0.05 (0.04–0.09), prorb = 0.72 (0.72–0.93), rcorb = 0.93 (0.92–1.06), vb = 0.71 (0.40–0.78), dcl = 0.24 (0.25–0.40), sctl = 0.99 (0.90–1.28), sterno = 0.61 (0.44–0.68), orbito = 1.20 (1.00–1.44), dcp = 0.20 (0.16–0.22), sctlp = 1.17 (1.05–1.30).

*Type specimens*: Holotype male (SCAU, no. 122235), CHINA: Hesong, Menghai, Yunnan, 1,600–1,900 m, 17.iv.2010, *ex.* tussock, JJ Gao. Paratypes: CHINA: seven males, five females (three males and two females in KIZ, nos. 0090548–552; four males and three females in SCAU, nos. 122236–42), *ex*. tussock and tree trunks, 16,17.iv.2010, JJ Gao, YR Su, L Wang, L Wu, same data as holotype; three males, one female (SCAU, nos. 122243–46), 7.iv.2011, JM Lu, SJ Yan, ZF Shao, YR Su, same data as holotype; five males, one female (SCAU, nos. 122247–52), 12.v.2012, HW Chen, same data as holotype.

*Etymology*: From the Latin world: amoenus, meaning delighted.

*Distribution*: China (Yunnan).

***Pseudostegana ximalaya* Zhang & Chen, sp. nov.**
urn:lsid:zoobank.org:act:3AE261B2-3982-42B3-ABC6-B62141244BD9
(Figs. 6A–6F and 15)

*Diagnosis*: This species differs from the other species of the *latiparma* group by having the epandrium with six strong prensisetae on ventral margin (Fig. 15A).

*Description*: Male and female. **Head**: Ocellar triangle brown on posterior three-quarter, dark brown on anterior a quarter (Fig. 6A). Frons brown (Fig. 6A). Face brown above, dark brown below. Clypeus black. Gena brown. Palpus dark brown, broadened in male, medially one-third as wide as long in male (Fig. 6D), brown in female. **Thorax**: Mesonotum brownish yellow anteriorly, yellowish brown to brown posteriorly (Fig. 6B); scutellum yellowish brown, yellow at tip (Fig. 6B). Pleura glossy, yellow on anterior one-third, dark brown on posterior two-thirds (Fig. 6E). **Legs**: Yellow. **Abdomen**: Tergites glossy, brownish yellow on second and third in male but only on second in female, the rest black (Fig. 6C). Sternites brownish to dark brown. **Male terminalia**: Surstylus strongly protruded on postero-ventral corners (Figs. 15A and 15B). Hypandrium with one pair of paramedian setae (Figs. 15C and 15D). Paramere dorsally protruded submedially, slender distally (Figs. 15C and 15D). Aedeagus dorsally curved apically in lateral view (Fig. 15D), with four finger-like processes basally (Figs. 15C and 15D).

*Measurements*: BL = 2.72 mm in holotype (range in three females paratypes: 2.25–2.83 mm), ThL = 1.08 mm (0.89–1.27 mm), WL = 2.09 mm (1.77–2.32 mm), WW = 0.95 mm (0.81–0.95 mm), arb = 6/1 (5–6/1), avd = 1.05 (0.93–0.98), adf = 1.64 (1.59–1.78), flw = 2.03 (1.52–1.66), FW/HW = 0.39 (0.40–0.46), ch/o = 0.05 (0.06–0.09), prorb = 0.80 (1.05–1.11), rcorb = 0.97 (0.90–1.01), vb = 0.72 (0.50–0.87), dcl = damaged (damaged), sctl = 1.13 (1.00–1.08), sterno = 0.62 (0.36–0.75), orbito = 1.02 (1.00–1.11), dcp = damaged (damaged), sctlp = 1.17 (1.11–1.26).

*Type specimens*: Holotype male (SCAU, no. 122256), CHINA: Beibeng, Motuo, Xizang, 800 m, 29.ix.2010, *ex.* tussock JJ Gao. Paratypes: CHINA: three females (SCAU, nos. 122257–59), *ex.* fallen logs, L Wang, L Wu, same data as holotype.

*Etymology*: The name means “Snow Country” in Tibetan, referring to the type locality.

*Distribution*: China (Xizang).

***Pseudostegana zhuoma* Zhang & Chen, sp. nov.**
urn:lsid:zoobank.org:act:835597CE-9D32-4928-96BC-CC3EEBBA760D
(Figs. 6G–6L and 16)

*Diagnosis*: This species is distinguished from *Pseudostegana amoena* sp. nov. by having the mesonotum brown, submedially with two pairs of yellow longitudinal stripes not reaching the scutellum (Fig. 6H); the pleura yellow on anterior quarter, brown on posterior 3/4 (Fig. 6K); the aedeagus ventrally protruded submedially in lateral view (Fig. 16C).

*Description*: Male and female. **Head**: Ocellar triangle narrowly elongated, yellow (Fig. 6G). Frons brown (Fig. 6G). Face brownish. Clypeus dark brown. Gena brown. Palpus yellow, slightly broadened in male, medially a quarter as wide as long (Fig. 6J), brown and slender in female. **Thorax**: Scutellum brown, yellow at tip (Fig. 6H). **Legs**: Yellow, brown on femur of foreleg, and knees of mid- and hindlegs. **Abdomen**: Tergites glossy, brown to dark brown, submedially with two yellow patches each on second and third tergites (Fig. 6I). Sternites brownish yellow. **Male terminalia**: Epandrium broadened on posterior margin, roundly protruded on ventral margin (Fig. 16A). Surstylus broadened, with several setae on outer surfaces (Fig. 16A). Hypandrial paramedian setae absent (Figs. 16B and 16C). Paramere round apically, with one acute dorsal projection; broadened subapically (Figs. 16B and 16C). Aedeagus broadways expanded apically, slightly dorsad protruded submedially in lateral view, with rod-like processes basally (Figs. 16B and 16C).

*Measurements*: BL = 2.90 mm in holotype (range in two females paratypes: 3.63–3.73 mm), ThL = 1.24 mm (1.49–1.50 mm), WL = 2.95 mm (3.21–3.37 mm), WW = 1.35 mm (1.61), arb = 6/1 (6/1), avd = 0.94 (0.90–1.08), adf = 1.41 (1.76–1.79), flw = 1.82 (2.05–2.10), FW/HW = 0.48 (0.59–0.60), ch/o = 0.07 (0.06–0.10), prorb = 0.87 (0.84–0.90), rcorb = 0.98 (0.87–0.88), vb = 0.78 (0.69–0.85), dcl = 0.28 (0.26–0.36), sctl = 0.83 (1.03–1.05), sterno = 0.70 (0.43–0.66), orbito = 0.90 (0.78–0.88), dcp = 0.13 (0.17–0.25), sctlp = 0.82 (0.75–0.97).

*Type specimens*: Holotype male (SCAU, no. 122253), CHINA: Tongmai, Bomi, Xizang, 30°06′N, 95°05′E, 2,000 m, 9.x.2010, *ex.* tussock, JJ Gao. Paratypes: CHINA: two females (SCAU, nos. 122254, 55), same data as holotype.

*Etymology*: The name means “fairy maiden” in the Tibetan, referring to the type locality.

*Distribution*: China (Xizang).

The *zonaria* species group

*Diagnosis*: Wing: Medial band at least as broad as length of dm-cu crossvein; r-m crossvein clear; M vein gently curved to R_4+5_ vein after dm-cu crossvein (Figs. 6R and 7L).

**Figure 17:**
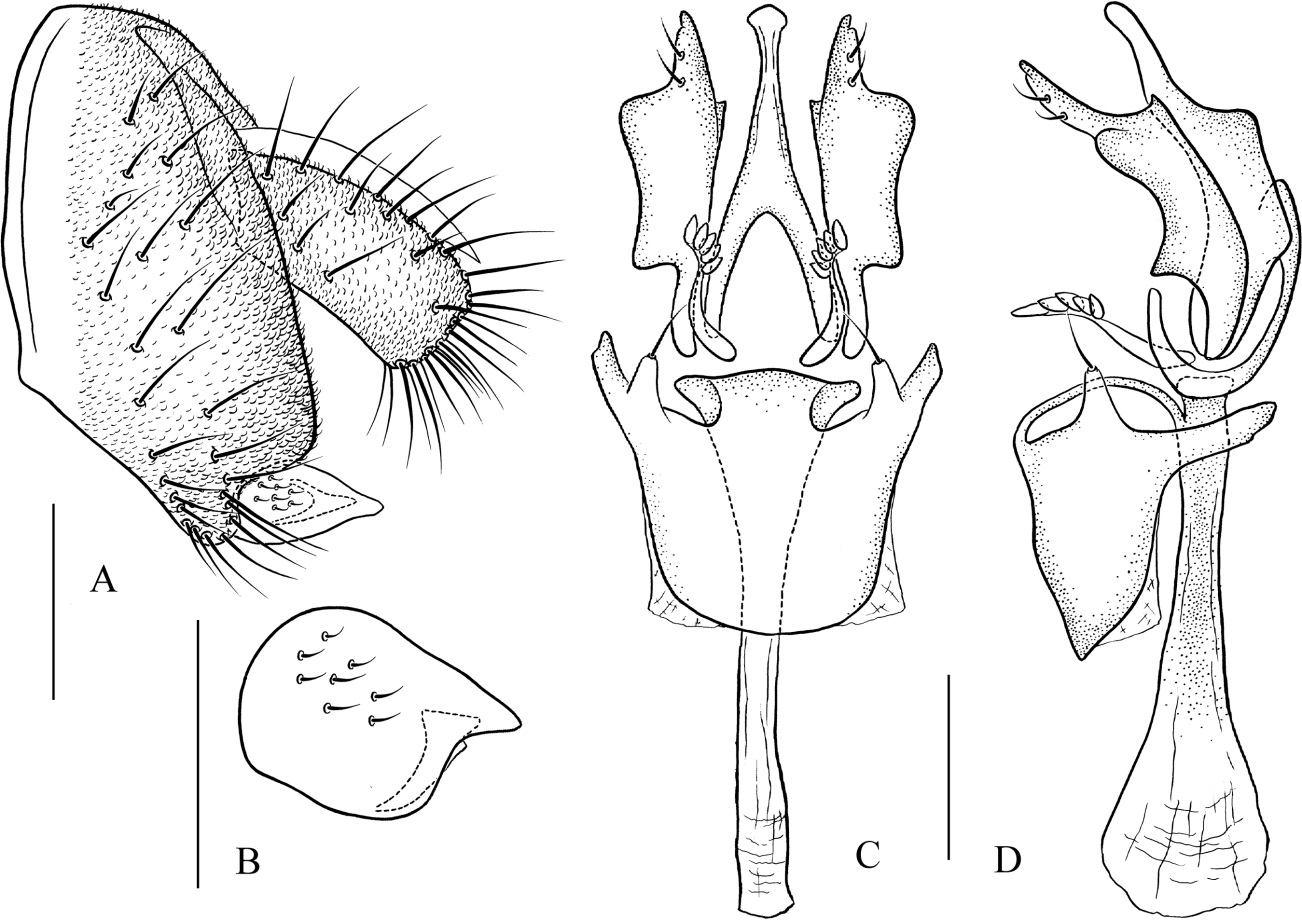
*Pseudostegana amnicola* sp. nov., male terminalia. (A) Epandrium, cercus and surstylus; (B) surstylus; (C) hypandrium, paramere, gonopods, aedeagus and aedeagal apodeme (ventral view); (D) ditto (lateral view). Scale bars = 0.1 mm. Drawing credit: Yuan Zhang.

***Pseudostegana amnicola* Zhang & Chen, sp. nov.**
urn:lsid:zoobank.org:act:17602786-17CA-4FD6-A6DB-5722E9FC5D72
(Figs. 6M–6R and 17)

*Diagnosis*: This species is similar to *Pseudostegana latifasciata* Chen, Toda & Wang, 2005 from Vietnam by the wing patches (Fig. 6R) and periphallic organs, but can be distinguished by the following characters: surstylus protruded on postero-ventral corner (Figs. 17A and 17B); aedeagus expanded apically (Fig. 17D). In *Pseudostegana latifasciata*: surstylus protruded on antero- and postero-ventral corner (Chen, Toda & Wang, 2005: Fig. 145); aedeagus slightly pointed apically in ventral view (Chen, Toda & Wang, 2005: Fig. 147).

*Description*: Male and female. **Head**: Ocellar triangle elongated (Fig. 6M). Frons brown (Fig. 6M). Face and gena brown. Clypeus black. Palpus brown, slender (Fig. 6P). **Thorax**: Mesonotum glossy, yellow on anterior half, brown (male, Fig. 6N) to black (female) on posterior half; scutellum brown in male (Fig. 6N), dark brown in female, pale at tip (Fig. 6N). Pleura glossy, brownish yellow on anterior half, brown (male, Fig. 6Q) to black (female) on posterior half. **Legs**: Yellow, black on mid- and hindlegs tibiae. **Abdomen**: All tergites glossy, dark brown in male (Fig. 6O), black in female. Sternites yellow in both sexes. **Male terminalia**: Epandrium roundly protruded on ventral margin, with numerous setae (Fig. 17A). Hypandrium with one pair of paramedian setae sublaterally (Figs. 17C and 17D). Paramere broadened subapically (Fig. 17C). Aedeagus with four finger-like processes basally (Figs. 17C and 17D).

*Measurements*: BL = 2.84 mm in holotype (range in four males and one females paratypes: 2.48–2.90 mm in males, 2.88 mm in females), ThL = 1.21 mm (0.97–1.30 mm in males, 1.25 mm in females), WL = 2.49 mm (2.15–2.67 mm in males, 2.53 mm in females), WW = 1.07 mm (0.97–1.23 mm in males, 1.05 mm in females), arb = 7/1 (5–8/1), avd = 0.97 (0.96–1.08), adf = 1.84 (1.50–1.90), flw = 1.75 (1.32–1.93), FW/HW = 0.48 (0.43–0.61), ch/o = 0.09 (0.05–0.07), prorb = 0.74 (0.85–1.00), rcorb = 0.91 (0.86–1.02), vb = 0.87 (1.00–1.42), dcl = damaged (damaged), sctl = 0.92 (1.08–1.18), sterno = 0.63 (0.61–0.74), orbito = 1.14 (1.11–1.23), dcp = 1.17 (1.20), sctlp = 1.02 (1.00–1.42).

*Type specimens*: Holotype male (SCAU, no. 122260), CHINA: Beibeng, Motuo, Xizang, 1,000 m, 1.x.2010, *ex.* fallen logs, JJ Gao. Paratypes: CHINA: one female (SCAU, no. 122261), same data as holotype; two males (SCAU, nos. 122262, 63), Baihualing, Baoshan, Yunnan, 25°18′N, 98°48′E, 1,400 m, 13.vi.2011, *ex.* tussock, JJ Gao, K Liu; one male (SCAU, no. 122159), Hesong, Menghai, Yunnan, 1,600 m, 13.v.2012, *ex.* tussock, HW Chen; one male (SCAU, no. 124884), Mengdong, Cangyuan, Yunnan, 23°10′N, 99°14′E, 1,320 m, 6.v.2016, *ex.* tussock, YQ Liu; two females (SCAU, nos. 111358, 59), Husa, Longchuan, Yunnan, 1,230 m, 20.viii.2016, *ex.* tussock, HW Chen, YQ Liu; one male (SCAU, no. 124842), Xincheng, Yingjiang, Yunnan, 960 m, 18,viii.2016, *ex.* tussock, L Gong.

*Etymology*: From the Latin word amnicola, meaning riverain.

*Distribution*: China (Yunnan).

**Figure 18:**
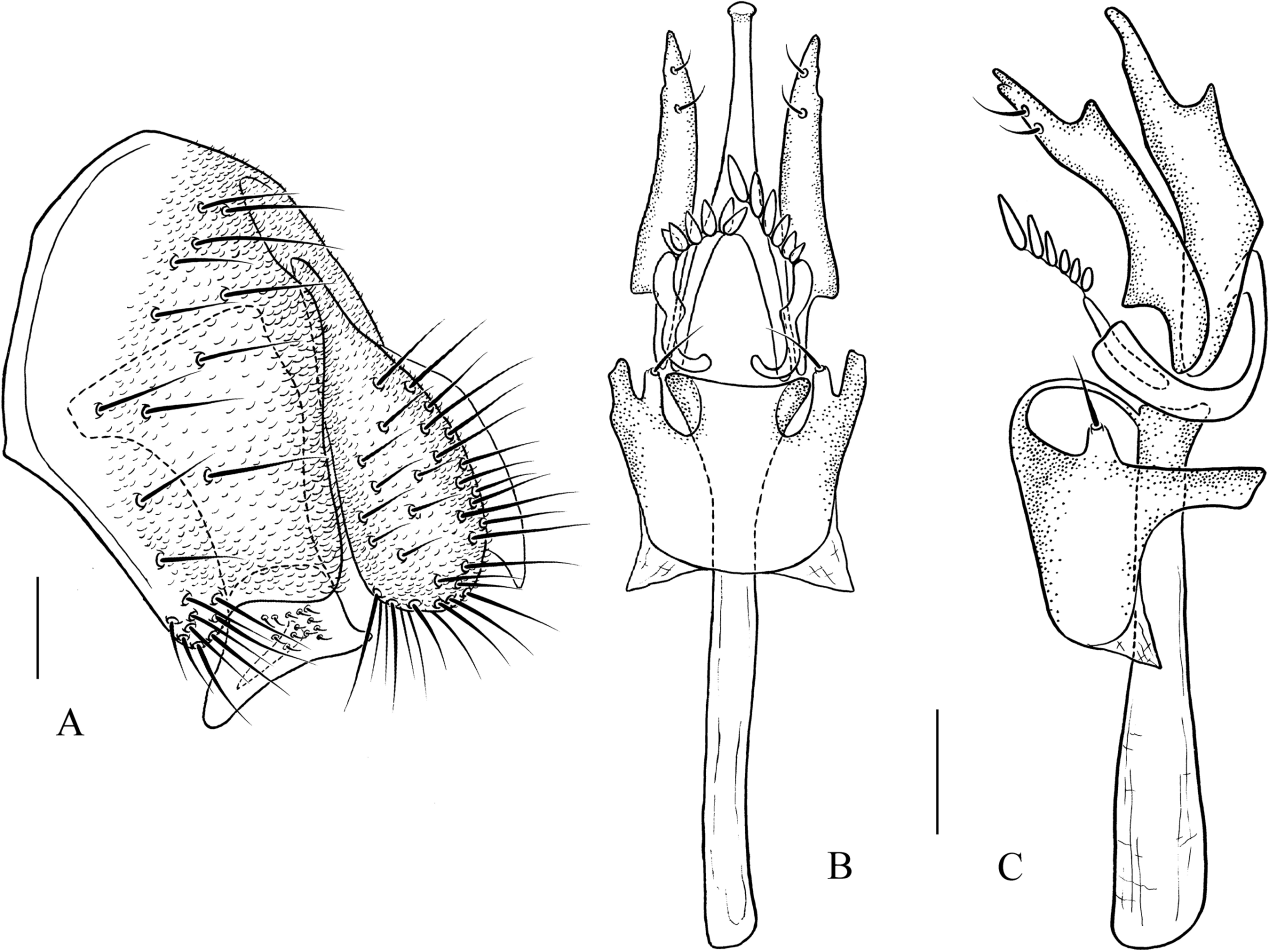
*Pseudostegana mailangang* sp. nov., male terminalia. (A) Epandrium, cercus and surstylus; (B) hypandrium, paramere, gonopods, aedeagus and aedeagal apodeme (ventral view); (C) ditto (lateral view). Scale bars = 0.1 mm. Drawing credit: Yuan Zhang.

***Pseudostegana mailangang* Zhang & Chen, sp. nov.**
urn:lsid:zoobank.org:act:048491D3-80F6-4F0C-A8EE-809113596DA9
(Figs. 7A–7F and 18)

*Diagnosis*: Mesonotum orange yellow on anterior half, dark brown to black on posterior half (Fig. 7B); paramere not broadened subapically in ventral view (Fig. 18B).

*Description*: Male and female. **Head**: Ocellar triangle elongated, dark brown (Fig. 7A). Frons, face and gena dark brown (Fig. 7A). Clypeus black. Palpus grayish brown, slender (Fig. 7D). **Thorax**: Mesonotum orange yellow on anterior half, dark brown to black on posterior half (Fig. 7B); scutellum dark brown, yellow at tip (Fig. 7B). Pleura glossy, mostly dark brown (Fig. 7E). **Legs**: Yellow, dark brown on femora and tibiae mid- and hindlegs. **Abdomen**: All tergites glossy, black (Fig. 7C). Sternites brown to dark. **Male terminalia**: Epandrium roundly protruded on ventral margin, with numerous setae (Fig. 18A). Surstylus protruded on antero- and postero-ventral corners (Fig. 18A); paramere smoothly narrowed in ventral view (Fig. 18B). Hypandrium with one pair of paramedian setae sublaterally (Figs. 18B and 18C). Aedeagus acutely protruded on distal one-third in lateral view (Fig. 18C), and with four finger-like processes basally (Figs. 18B and 18C).

*Measurements*: BL = 2.83 mm in holotype (range in five males and three females paratypes: 2.62–2.83 mm in males, 3.12–3.21 mm in females), ThL = 0.97 mm (0.70–1.05 mm in males, 1.19–1.25 mm in females), WL = 1.79 mm (1.87–2.08 mm in males, 2.19–2.33 mm in females), WW = 0.84 mm (0.83–0.93 mm in males, 1.03–1.07 mm in females), arb = 7/1 (6–9/1), avd = 0.94 (0.88–1.13), adf = 1.78 (0.88–2.38), flw = 1.43 (1.13–1.95), FW/HW = 0.40 (0.40–0.45), ch/o = 0.05 (0.04–0.07), prorb = 1.08 (0.81–1.12), rcorb = 0.98 (0.82–0.95), vb = 1.04 (0.69–1.14), dcl = damage (0.29–0.38), sctl = 1.25 (0.91–1.25), sterno = 0.57 (0.43–0.62), orbito = 1.07 (1.06–1.29), dcp = 0.25 (0.19–0.24), sctlp = 1.31 (1.13–1.73).

*Type specimens*: Holotype male (SCAU, no. 122264), CHINA: Wangtianshu, Mengla, Yunnan, 21°28′N, 101°38′E, 600 m, 31.ix.2011, *ex.* fallen logs, HW Chen. Paratypes: CHINA: seven males, three females (two males and one female in KIZ, nos. 0090507–09; five males and two females in SCAU, nos. 122265–71), HW Chen, JJ Gao, same data as holotype; one male (SCAU, no. 124885), Guanlei, Mengla, Yunnan, 21°38′N, 101°10′E, 620 m, 23.iv.2016, *ex*. tussock, YQ Liu.

*Etymology*: The name means “tall tree” in the language of the Dai nationality in Yunnan Province.

*Distribution*: China (Yunnan).

**Figure 19:**
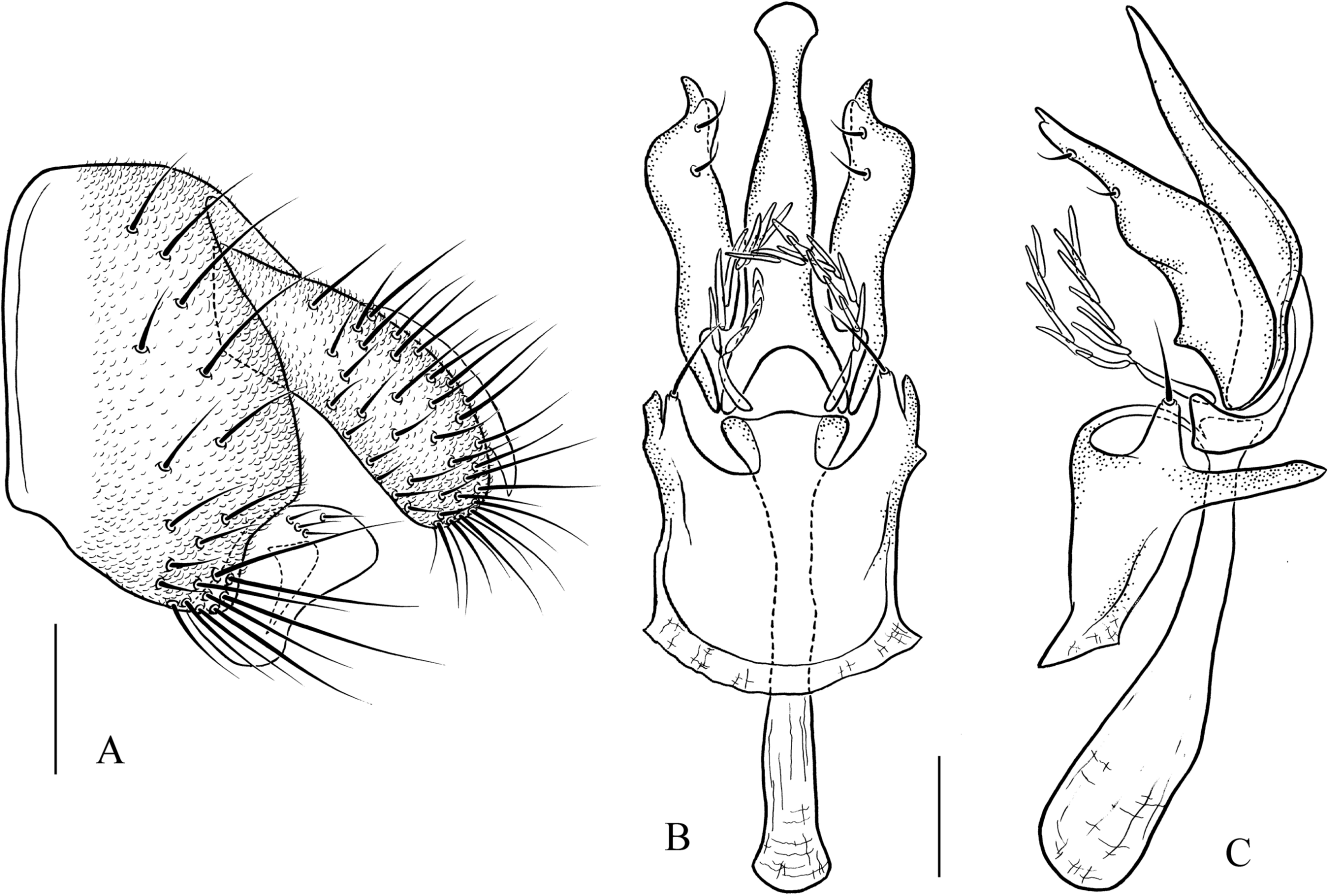
*Pseudostegana mystica* sp. nov., male terminalia. (A) Epandrium, cercus and surstylus; (B) hypandrium, paramere, gonopods, aedeagus and aedeagal apodeme (ventral view); (C) ditto (lateral view). Scale bars = 0.1 mm. Drawing credit: Yuan Zhang.

***Pseudostegana mystica* Zhang & Chen, sp. nov.**
urn:lsid:zoobank.org:act:7754C40D-A608-48F3-AACF-431F3E5F8D9D
(Figs. 7G–7L and 19)

*Diagnosis*: This species is similar to *Pseudostegana insularis* Li, Gao & Chen, 2010 from Hainan, China by the wing patches (Fig. 7L) and periphallic organs (Fig. 19A), but can be distinguished by the aedeagus being broadened apically (Fig. 19B).

*Description*: Male. **Head**: Ocellar triangle elongated, yellowish (Fig. 7G). Frons and gena yellow (Fig. 7G). Face mostly yellow, brown on lower margin. Clypeus brown. Palpus yellow, slender (Fig. 7J). **Thorax**: Mesonotum mostly yellow; scutellum brown, yellow at tip (Fig. 7H). Pleura glossy, yellow on anterior two-thirds, brown on posterior one-third (Fig. 7K). **Legs**: Mostly yellow. **Abdomen**: All tergites glossy, mostly black (Fig. 7I). Sternites yellow. **Male terminalia**: Epandrium slightly roundly protruded on ventral margin, with numerous setae (Fig. 19A). Surstylus protruded on antero- and postero-ventral corners (Fig. 19A). Hypandrium with one pair of paramedian setae sublaterally (Figs. 19B and 19C). Aedeagus smoothly narrowed on distal two-thirds, with seven finger-like process basally (Figs. 19B and 19C).

*Measurements*: BL = 2.40 mm in holotype, ThL = 1.25 mm, WL = 2.63 mm, WW = 1.10 mm, arb = 7/1, avd = 0.87, adf = 1.88, flw = 2.11, FW/HW = 0.39, ch/o = 0.07, prorb = 0.83, rcorb = 0.83, vb = 0.72, dcl = 0.27, sctl = damaged, sterno = 0.53, orbito = 1.26, dcp = 0.19, sctlp = 1.00.

*Type specimen*: Holotype male (SCAU, no. 122272), CHINA: Beibeng, Motuo, Xizang, 1,000 m, 1.x.2010, *ex.* fallen logs, JJ Gao.

*Etymology*: From the Latin word silvanus, meaning forest deity.

*Distribution*: China (Xizang).

### Key to all Chinese species of the genus *Pseudostegana*

1. Wing with distinct cross band subbasally or CuA_1_ vein subbasally with distinct patch below (Figs. 4F, 4L, 4R, 5F, 5L, 5R, 6F and 6L)2
  – Wing neither with distinct cross band subbasally nor CuA_1_ vein subbasally with distinct patch below (Figs. 6R, 7F and 7L) (*zonaria* group)16
2. Wing subbasal and medial, cross bands separated from each other (Figs. 4F, 5L, 5R, 6F and 6L)3
  – Wing subbasal and medial, cross bands confluent posteriorly, forming V-shaped pattern (Figs. 4L, 4R and 5F) (*javana* group)4
3. Ocellar triangle not elongated (Fig. 4A); M vein strongly curved after dm-cu crossvein (Fig. 4F); wing with annular patch medially (Fig. 4F) (*fleximediata* group) *Pseudostegana meiduo* sp. nov.
  – Ocellar triangle elongated (Figs. 5A, 5G and 6A); M vein gently curved after dm-cu crossvein; wing with only one medial band (Figs. 5L, 5R, 6F and 6L) (*latiparma* group)7
4. Epandrium strongly roundly protruded ventrally, with dense setae (Fig. 11A); aedeagus apically slightly concave in ventral view (Fig. 11C) *Pseudostegana stictiptrata* sp. nov.
  – Epandrium slightly protruded ventrally (Figs. 10A and 12A); aedeagus apically slightly protruded in ventral view (Figs. 10C and 12C)5
5. Mesonotum orange yellow on anterior one-third, black on posterior two-thirds; scutellum orange yellow, black on lateral margins Pseudostegana xanthoptera
  – Mesonotum yellow to yellowish brown (Figs. 4H and 5B); scutellum yellow (Figs. 4H and 5B)6
6. Surstylus strongly protruded on posterior corner in lateral view, broadened, approximately 1.5 times as high as wide (Figs. 10A and 10B)*Pseudostegana meiji* sp. nov.
  – Surstylus strongly protruded and pointed on anterior corner, broadened, approximately two times as high as wide (Figs. 12A and 12B) *Pseudostegana stigmatptera* sp. nov.
7. R-m crossvein clouded; medial, dark-color band much broadened and with one distinct, protruded part submedially (Li, Gao & Chen, 2010: Fig. 15) Pseudostegana bilobata
  – R-m crossvein clear (Figs. 5L, 5R, 6F and 6L)8
8. Palpus expanded, medially one-third to half as wide as long (Fig. 6D)9
  – Palpus slender, rod-shaped11
9. Epandrium with six strong prensisetae on ventral margin (Fig. 15A)*Pseudostegana ximalaya* sp. nov.
  – Epandrium without strong setae on ventral margin10
10. Paramere apically shallowly bifurcated, subapically lacking small projection, dorsomedially slightly roundly protruded in lateral view (Chen, Toda & Wang, 2005: Fig. 93) Pseudostegana bifasciata
  – Paramere apically not bifurcated, subapically with one small, acute projection, dorsomedially triangularly protruded in lateral view (Li, Gao & Chen, 2010: Figs. 3D and 3E) Pseudostegana acutifoliolata
11. Mesonotum with pattern (Figs. 5N and 6H)12
  – Mesonotum with one brown longitudinal stripe at most (Figs. 5H, 8D, 8M and 8P)13
12. Mesonotum yellow, with five brown longitudinal stripes (Fig. 5N); abdominal second and third tergites mostly yellow (Fig. 5O) *Pseudostegana amoena* sp. nov.
  – Mesonotum brown, submedially with two pairs of yellow longitudinal stripes (Fig. 6H); abdominal second and third tergites each with two yellow patches submedially (Fig. 6I) *Pseudostegana zhuoma* sp. nov.
13. Paramere subapically with one acute, dorsal projection (Fig. 13A)14
  – Paramere subapically without projection.15
14. Mesonotum antero-medially yellow and with thin brownish yellow, longitudinal stripe, posteriorly and laterally brownish yellow (Fig. 5H); aedeagus apically pointed in ventral view (Fig. 13D) *Pseudostegana alpina* sp. nov.
  – Mesonotum yellow on anterior one-third, brown to black on posterior two-thirds (Fig. 8M); aedeagus hammer-shaped in lateral view (Li, Gao & Chen, 2010: Fig. 7E) Pseudostegana minutipalpata
15. Paramere subapically triangularly expanded in ventral view; basal process of aedeagus membranous, lacking finger-like processes (Chen, Toda & Wang, 2005: Figs. 92 and 93) Pseudostegana angustifasciata
  – Paramere subapically roundly expanded in ventral view; basal process of aedeagus with finger-like processes (Chen, Toda & Wang, 2005: Figs. 105 and 106) Pseudostegana pallidimaculata
16. Palpus expanded, medially wider than one-third of length17
  – Palpus slender, rod-like.19
17. Paramere apically bifurcated (Li, Gao & Chen, 2010: Fig. 8E) Pseudostegana insularis
  – Paramere apically not bifurcated (Li, Gao & Chen, 2010: Fig. 9E)18
18. Palpus black, yellow at tip (Li, Gao & Chen, 2010: Fig. 1L); aedeagus strongly narrowed on distal one-third Pseudostegana silvana
  – Palpus yellow; aedeagus smoothly narrowed on distal half Pseudostegana latipalpis
19. Paramere with one nearly triangular process on distal one-third in lateral view20
  – Paramere distally lacking process in lateral view21
20. Paramere not broadened subapically in ventral view (Fig. 18B); aedeagus triangularly protruded on distal one-third in lateral view (Fig. 18C) *Pseudostegana mailangang* sp. nov.
  – Paramere broadened subapically in ventral view (Fig. 17C); aedeagus slightly roundly protruded on distal one-third in lateral view (Fig. 18D) *Pseudostegana amnicola* sp. nov.
21. Paramere not bifurcated apically (Chen, Toda & Wang, 2005: Fig. 144); aedeagus slightly pointed in ventral view (Chen, Toda & Wang, 2005: Fig. 143) Pseudostegana dolichopoda
  – Paramere shallowly bifurcated apically (Figs. 19B and 19C); aedeagus apically slightly expanded22
22. Aedeagus apically heart-shaped in ventral view, protruded on distal one-third in lateral view (Chen, Toda & Wang, 2005: Figs. 149 and 150) Pseudostegana nitidifrons
  – Aedeagus apically roundly expanded in ventral view, nearly straight in lateral view (Figs. 19B and 19C) *Pseudostegana mystica* sp. nov.

## Discussion

DNA sequence data may process effective species boundary information, which provides a useful tool for taxonomic studies (Hamilton et al., 2014; Kekkonen et al., 2015). The integration of DNA sequence data and the traditional morphological characters increases the ease and reliability of both species identification and species discovery (Vogler, 2006; Cardoso, Serrano & Vogler, 2009; Kekkonen & Hebert, 2014; Roberts et al., 2016).

We proposed the *Pseudostegana* species based on morphological variation and then clarified their status by DNA data. Although some new species are described (e.g., *Pseudostegana alpina* sp. nov. and *Pseudostegana meiduo* sp. nov.) based on few observed specimens, both morphological and molecular analysis support our taxonomic hypothesis. The new species, *Pseudostegana alpina* sp. nov., *Pseudostegana amoena* sp. nov., *Pseudostegana mailangang* sp. nov., *Pseudostegana meiduo* sp. nov., *Pseudostegana meiji* sp. nov., *Pseudostegana stictiptrata* sp. nov., *Pseudostegana stigmatptera* sp. nov., *Pseudostegana ximalaya* sp. nov. and *Pseudostegana zhuoma* sp. nov., which we described here, were recovered as distinct entities in phylogenetic trees and the ABGD analyses. The minimal interspecific genetic divergences between these new species and their close relatives all exceed 3%. DNA data support the morphological characteristics observed among these nine Chinese species and confirms the new species as being distinctly different.

*Pseudostegana mystica* sp. nov. from Xizang is similar to *Pseudostegana insularis* from Hainan in the wing patches, but they can be morphologically distinguished by the aedeagus, which is apically expanded in ventral view in the former one (Li, Gao & Chen, 2010). Although we only amplified the *COI* region for *Pseudostegana mystica* sp. nov., the *COI* marker still provided reliable evidence for its new species status, as high genetic divergences (>14%; Table S3) were observed between *Pseudostegana mystica* sp. nov. and the other *Pseudostegana* species.

High intraspecific variation is sometimes observed among Drosophilidae species, for example, estimates of *COI* intraspecific divergence were 8%, 9% and 11% for three *Drosophila* species (Yassin et al., 2010), and 4.4% for the *Leucophenga euryphylla* (Huang et al., 2013). Interestingly, the Yunnan lineage and Xizang lineage of *Pseudostegana amnicola* sp. nov. show relatively large sequence divergences (5.5% for *COI*, 5.1% for *ND2*). Moreover, the ABGD analysis based on *COI* data separated them to two MOTUs. Although no obvious morphological variation was observed, genetic diversification seems to have occurred between these two geographic lineages. The prominent genetic divergence between lineages probably indicate the presence of unrecognized cryptic species. More samples from different populations and genders should be sampled in order to assess if they belong to cryptic species or not.

The *COI* and *ND2* genes are useful for species identification or discrimination of Drosophilidae species (He et al., 2009; Zhao, Gao & Chen, 2009; Lu et al., 2011; Lu, Li & Chen, 2011; Zhao et al., 2013; Shao et al., 2014; Zhang, Li & Chen, 2016). Our results show that *COI* barcoding successfully distinguished all the studied *Pseudostegana* species and helped to discover the potential new species. The power of the *ND2* fragment to discriminate the studied specimens of *Pseudostegana* at the species level is similar to that of the *COI* fragment. In several studies, the nuclear *28S* gene was successful for identification in insect at the species level (Monteiro et al., 2000; Monaghan et al., 2005; Asokan et al., 2013; Resch et al., 2014). In contrast to *COI* and *ND2*, the *28S* fragment showed less resolution at species level for the studied *Pseudostegana*. This can possibly be attributed to the conservation of the *28S* gene such that closely related species showed little variation in sequences.

Chen, Toda & Wang (2005) examined 32 *Pseudostegana* species, and proposed six species groups (the *atrofrons*, *javana*, *latiparma*, *grandipalpis*, *fleximediata* and *zonaria* groups) on the basis of morphological features. In our phylogeny, *Pseudostegana meiduo* sp. nov. is placed at the basal position within *Pseudostegana* and recovered as a distinct clade from the other three species groups (the *latiparma*, *java* and *zonaria* groups). Morphologically, *Pseudostegana meiduo* sp. nov. is classified in the *fleximediata* group as it is congruent with the description of the species group, e.g., ocellar triangle is not elongated and M vein is strongly curved after dm-cu crossvein (Fig. 4F in Chen, Toda & Wang, 2005). However, its wing pattern differs from the other *fleximediata* group species by having distinct cross band at the basal position of the wing. Unfortunately, no other *fleximediata* group species was included in the present phylogenetic analyses for testing its phylogenetic status. We temporarily place *Pseudostegana mediuo* sp. nov. in the *fleximediata* group, but its taxonomic status should be evaluated by additional sampling. Phylogenetic analyses recovered the monophyly of the *latiparma* and *javana* groups, but the *zonaria* group was found to be paraphyletic as clade I (*Pseudostegana insularis* + *Pseudostegana silvana*) were separated from clade II (*Pseudostegana nitidifrons* + *Pseudostegana amnicola* sp. nov. + *Pseudostegana mailangang* sp. nov.). The latter clade is morphologically different from the former concerning the shape of the palpus. The palpus of *Pseudostegana latifasciata*, *Pseudostegana nitidifrons*, *Pseudostegana amnicola* sp. nov. and *Pseudostegana mailangang* sp. nov. are slender and rod-like shape, while the palpus of the other *zonaria* group species are expanded (Chen, Toda & Wang, 2005).

Yunnan and the adjacent area (Southwest China) are located at the junction of the Himalaya, Mountains of Southwest China and Indo-Burma biodiversity hotspots (Myers et al., 2000). In recent years, fieldwork by members of our laboratory have revealed a hidden diversity of *Pseudostegana* species in this area, where previously only eight species were reported (Chen, Toda & Wang, 2005; Li, Gao & Chen, 2010). Southwest China total contains 38% (19 out of 50; including new species described in this study) of *Pseudostegana* species, and all of them are endemic to this region. Southwest China seems to be an important center of diversification of *Pseudostegana* species. Moreover, the short, relatively ancient branches of the phylogenetic trees suggest that adaptive radiation probably occurred during the early evolutionary history of *Pseudostegana*. Although we provided a molecular phylogeny for the Chinese *Pseudostegana*, additional research on systematics including other species groups and species in Southwest Asia, will be needed to better understand the origin and diversification of this genus.

## Supplemental Information

10.7717/peerj.5160/supp-1 Supplemental Information 1 Fig. S1. Bayesian tree of the genus *Pseudostegana* inferred from the *COI*, *ND2* and *28S* sequences.

10.7717/peerj.5160/supp-2 Supplemental Information 2 Table S1. Primers used for PCR and sequencing in this study.

10.7717/peerj.5160/supp-3 Supplemental Information 3 Table S2. The definitions of measurements and indices of Zhang & Toda (1994) and Chen & Toda (2001).

10.7717/peerj.5160/supp-4 Supplemental Information 4 Table S3. Summary of intra- and interspecific genetic distances of *COI* region.

10.7717/peerj.5160/supp-5 Supplemental Information 5 Table S4. Summary of intra- and interspecific genetic distances of *ND2* region.

10.7717/peerj.5160/supp-6 Supplemental Information 6 Table S5. Summary of intra- and interspecific genetic distances of* 28S* region.

10.7717/peerj.5160/supp-7 Supplemental Information 7 Table S6. The ABGD analysis result based on the *COI* data set.

10.7717/peerj.5160/supp-8 Supplemental Information 8 Table S7. The ABGD analysis result based on the *ND2* data set.

10.7717/peerj.5160/supp-9 Supplemental Information 9 Table S8. The ABGD analysis result based on the *COI* data set.

## Additional Information and Declarations

### Data Availability

The following information was supplied regarding data availability: GenBank: KP981250–KP981252; KY542040–KY542046; KY542049–KY542050; KY542053; KY542055–KY542066; KY542068–KYK52077; KY542080; KJ813837–KJ813969; KJ813971–KJ814001; KJ813904–KJ813935.

### New Species Registration

The following information was supplied regarding the registration of a newly described species: Publication LSID: urn:lsid:zoobank.org:pub:91F288F7-0083-4EBD-9B49-DF51048E9519; Genus name, *Pseudostegana*: urn:lsid:zoobank.org:act:C447025C-6DCC-4D88-A5B1-095925763035, Species name: urn:lsid:zoobank.org:act:CD5E03C7-3130-4FF6-9A1B-C4FE6690B7ED, urn:lsid:zoobank.org:act:0E0F9E07-C2E7-48F6-B021-F699E21F88CC, urn:lsid:zoobank.org:act:88C101ED-F994-4E0A-8CCB-B2F60378412F, urn:lsid:zoobank.org:act:72488FCE-4052-403B-A450-3A9312B4BB2F, urn:lsid:zoobank.org:act:31D32207-72FD-41A4-8971-8349404EB97C, urn:lsid:zoobank.org:act:D3696645-FA69-4B4B-A5D8-D6FCF6F90764, urn:lsid:zoobank.org:act:3AE261B2-3982-42B3-ABC6-B62141244BD9, urn:lsid:zoobank.org:act:835597CE-9D32-4928-96BC-CC3EEBBA760D, urn:lsid:zoobank.org:act:17602786-17CA-4FD6-A6DB-5722E9FC5D72, urn:lsid:zoobank.org:act:048491D3-80F6-4F0C-A8EE-809113596DA9, urn:lsid:zoobank.org:act:7754C40D-A608-48F3-AACF-431F3E5F8D9D.
