# Supplementary figures and images for: Integrative taxonomy of the genus Pseudostegana (Diptera, Drosophilidae) from China, with descriptions of eleven new species
Source: PeerJ. 2018 Sep 5;6:e5160. doi: 10.7717/peerj.5160 (PMC6129143; doi:10.7717/peerj.5160)

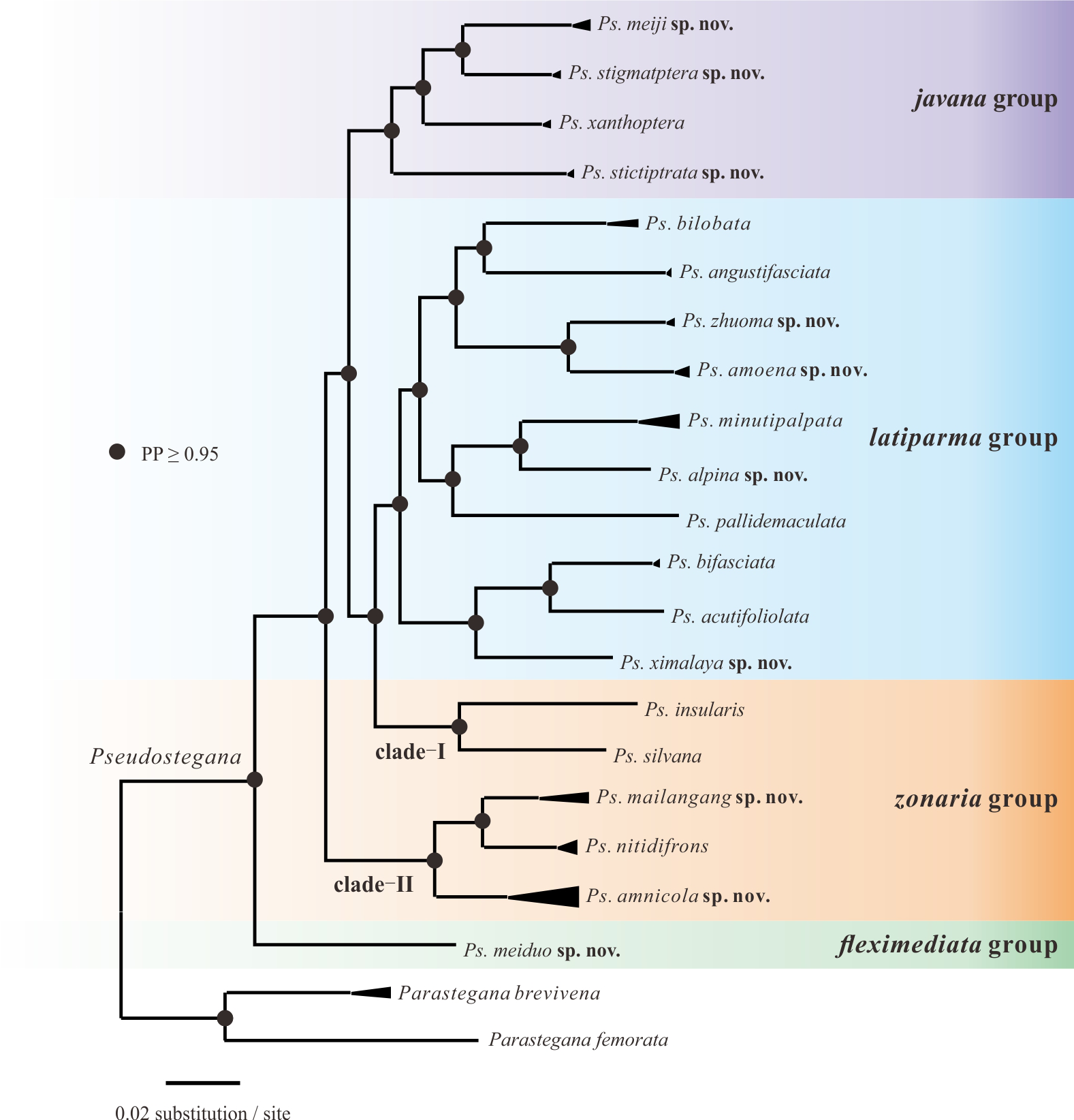

Supplement: Supplemental Information 1 [file peerj-06-5160-s001.png]
