# Supplementary material for: Integrative taxonomy of the genus Pseudostegana (Diptera, Drosophilidae) from China, with descriptions of eleven new species
Source: PeerJ. 2018 Sep 5;6:e5160. doi: 10.7717/peerj.5160 (PMC6129143; doi:10.7717/peerj.5160)
Supplement: Supplemental Information 2 [file peerj-06-5160-s002.docx]

Table S1. Primers used for PCR and sequencing in this study.

| Primer name | F/R* | Primer sequence (5’--3’) | Utility | References |
| --- | --- | --- | --- | --- |
| *COI*-F1 | F | CGCCTAAACTTCAGCCACTT | PCR/sequencing | He et al., 2009 |
| *COI*-F2 | F | ATCGCCTAAACTTCAGCCAC | PCR/sequencing | Wang et al., 2006 |
| *COI*-R1 | R | CCTAAATTAGCTCATGTAGAC | PCR/sequencing | He et al., 2009 |
| *COI*-R2 | R | TCCATTGCACTAATCTGCCA | PCR/sequencing | Wang et al., 2006 |
| HCO2198 | R | TAAACTTCAGGGTGACCAAAAAATCA | PCR/sequencing | Folmer et al., 1994 |
| *ND2*-H | F | AAGCTACTGGGTTCATACC | PCR/sequencing | Park, 1999 |
| *ND2*-T1 | R | ATATTTACAGCTTTGAAGG | PCR/sequencing | Park, 1999 |
| *ND2*-T2 | R | GCTTTGAAGGCTATTAGTT | PCR/sequencing | He et al., 2009 |
| *28S*-F1 | F | GACTACCCCCTGAATTTAAGCAT | PCR/sequencing | Kim et al., 2000 |
| *28S*-F2 | F | CAACCTCAACTCATATGGGAC | PCR/sequencing | Cao et al., 2011 |
| *28S*-R1 | R | CCATCTTTCGGGTCACAGCAT | PCR/sequencing | Cao et al., 2011 |
| *28S*-R2 | R | GTTCACCATCTTTCGGGTCA | PCR/sequencing | Cao et al., 2011 |

* F and R represent forward and reserved primers, respectively.

**References:**

**Cao HL, Wang XL, Gao JJ, Stéphane RP, Watabe HA, Zhang YP, Chen HW.** **2011.** Phylogeny of the African and Asian *Phortica* (Drosophilidae) deduced from nuclear and mitochondrial DNA Sequences. *Molecular Phylogenetics and Evolution* **61:** 677–685.

**Folmer O, Black M, Hoeh W, Lutz R, Vrijenhoek R. 1994.** DNA primers for amplification of mitochondrial cytochrome c oxidase subunit I from diverse metazoan invertebrates. *Molecular Marine Biology and Biotechnology* **3:** 294–299.

**He XF, Gao JJ, Cao HZ, Zhang XL, Chen HW. 2009.** Taxonomy and molecular phylogeny of the *Phortica hani* species complex (Diptera: Drosophilidae). *Zoological Journal of the Linnean Society* **157:** 359–372.

**Kim CG, Zhou HZ, Imura Y, Tominaga O, Su ZH, Osawa S. 2000.** Pattern of morphological diversification of the *Leptocarabus* ground beetles as deduced from mitochondrial *ND5* gene and nuclear *28S* rDNA sequences. *Molecular Biology and Evolution* **17:** 137–145.

**Park J. 1999.** Molecular phylogenetic studies of the *Drosophila* (*Drosophila*) *virilis* section (Diptera, Drosophilidae). PhD. Thesis, Tokyo Metropolitan University.

**Wang BC, Park J, Watabe HA, Gao JJ, Xiangyu JG, Aotsuka T, Chen HW, Zhang YP. 2006.** Molecular phylogeny of the *Drosophila virilis* section (Diptera: Drosophilidae) based on mitochondrial and nuclear sequences. *Molecular Biology and Evolution* **40:** 484–500.
