# Supplementary material for: Integrative taxonomy of the genus Pseudostegana (Diptera, Drosophilidae) from China, with descriptions of eleven new species
Source: PeerJ. 2018 Sep 5;6:e5160. doi: 10.7717/peerj.5160 (PMC6129143; doi:10.7717/peerj.5160)
Supplement: Supplemental Information 3 [file peerj-06-5160-s003.docx]

Table S2. The definitions of measurements and indices of Zhang & Toda (1994) and Chen & Toda (2001).

| Abbreviation | Definitions |
| --- | --- |
| BL | Body Length = straight distance from distal endge of pedicel to tip of abdomen |
| ThL | Thorax Length = distance from anterior notal margin to apex of scutellum |
| WL | Wing Length = distance from humeral crossvein to wing apex |
| WW | maximum wing width |
| HW | Head Width = greatest distance between apical portions of eyes |
| FW | Frontal Width = distance between eyes measured through anterior ocellus |
| arb | dorsal branches/ventral branches of arista |
| avd | longest ventral branch/longest dorsal branch of arista in length |
| adf | longest dorsal branch of arista/width of first flagellomere |
| flw | length/width of first flagellomere |
| FW/HW | frontal width/head width |
| ch/o | maximum width of gena/maximum diameter of eye |
| prorb | proclinate orbital/posterior reclinate orbital in length |
| rcorb | anterior reclinate orbital/posterior reclinate orbital in length |
| vb | subvibrissal/vibrissa in length |
| dcl | anterior dorsocentral/posterior dorsocentral in length |
| prescl | prescutellar/posterior dorsocentral in length |
| sctl | basal scutellar/apical scutellar in length |
| sterno | anterior katepisternal/posterior katepisternal in length |
| orbito | distance between proclinate and posterior reclinate orbitals/distance between inner vertical and posterior reclinate orbital |
| dcp | length distance between ipsilateral dorsocentrals/ cross distance between anterior |
| sctlp | distance between ipsilateral scutellars/cross distance between apical scutellars |
| C | second costal section between subcostal break and R2+3/third costal section between R2+3 and R4+5 |
| 4c | third costal section between R2+3 and R4+5/M1 between r-m and dm-cu |
| 4v | M1 between dm-cu and wing margin/M1 between r-m and dm-cu |
| 5x | CuA1 between dm-cu and wing margin/dm-cu between M1 and CuA1 |
| ac | third costal section between R2+3 and R4+5/distance between distal ends of R4+5 and M1 |
| M | CuA1 between dm-cu and wing margin/M1 between r-m and dm-cu |
| C3F | length of heavy setation in third costal section/length of heavy setation in third costal section + length of light setation in third costal section |

**References:**

**Chen HW, Toda MJ. 2001**. A revision of the Asian and European species in the subgenus *Amiota* Loew (Diptera: Drosophilidae) and establishment of species-groups based on phylogenetic analysis. *Journal of Natural History* **35**: 1517–1563.

**Zhang WX, Toda MJ. 1992**. A new species-subgroup of the *Drosophila immigrans* species-group (Diptera, Drosophilidae), with description of two new species from China and revision of taxonomic terminology. *Japanese Journal of Entomology* **60**: 839–850.
