# Supplementary material for: Integrative taxonomy of the genus Pseudostegana (Diptera, Drosophilidae) from China, with descriptions of eleven new species
Source: PeerJ. 2018 Sep 5;6:e5160. doi: 10.7717/peerj.5160 (PMC6129143; doi:10.7717/peerj.5160)
Supplement: Supplemental Information 4 [file peerj-06-5160-s004.docx]

Table S3. Summary of intra- and interspecific genetic distances of *COI* region.

|  | Species | N | intra | inter | | | | | | | | | | | | | | | | | | | |  |
| --- | --- | --- | --- | --- | --- | --- | --- | --- | --- | --- | --- | --- | --- | --- | --- | --- | --- | --- | --- | --- | --- | --- | --- | --- |
|  |  |  |  | (1) | (2) | (3) | (4) | (5) | (6) | (7) | (8) | (9) | (10) | (11) | (12) | (13) | (14) | (15) | (16) | (17) | (18) | (19) | (20) | (21) |
| (1) | *Ps. meiduo* **sp. nov.** | 1 | - | - |  |  |  |  |  |  |  |  |  |  |  |  |  |  |  |  |  |  |  |  |
| (2) | *Ps. xanthoptera* | 3 | 0.009-0.026 | 0.142-0.153 | - |  |  |  |  |  |  |  |  |  |  |  |  |  |  |  |  |  |  |  |
| (3) | *Ps. meiji* **sp. nov.** | 3 | 0.011-0.015 | 0.157-0.163 | 0.098-0.105 | - |  |  |  |  |  |  |  |  |  |  |  |  |  |  |  |  |  |  |
| (4) | *Ps. stictiptrata* **sp. nov.** | 2 | 0.009 | 0.144-0.148 | 0.130-0.147 | 0.105-0.116 | - |  |  |  |  |  |  |  |  |  |  |  |  |  |  |  |  |  |
| (5) | *Ps. stigmatptera* **sp. nov.** | 3 | 0.002-0.012 | 0.150-0.155 | 0.093-0.102 | 0.086-0.095 | 0.126-0.132 | - |  |  |  |  |  |  |  |  |  |  |  |  |  |  |  |  |
| (6) | *Ps. acutifoliolata* | 1 | - | 0.161 | 0.156-0.163 | 0.161-0.165 | 0.132-0.134 | 0.137-9.143 | - |  |  |  |  |  |  |  |  |  |  |  |  |  |  |  |
| (7) | *Ps. angustifasciata* | 2 | 0.000 | 0.152 | 0.148-0.152 | 0.147-0.154 | 0.143-0.147 | 0.143-0.145 | 0.160 | - |  |  |  |  |  |  |  |  |  |  |  |  |  |  |
| (8) | *Ps. bifasciata* | 2 | 0.006 | 0.146-0.150 | 0.151-0.168 | 0.152-0.164 | 0.139-0.152 | 0.139-0.149 | 0.105 | 0.153 | - |  |  |  |  |  |  |  |  |  |  |  |  |  |
| (9) | *Ps. bilobata* | 2 | 0.036 | 0.157-0.165 | 0.130-0.142 | 0.127-0.136 | 0.126-0.137 | 0.123-0.141 | 0.143-0.145 | 0.127-0.137 | 0.134-0.153 | - |  |  |  |  |  |  |  |  |  |  |  |  |
| (10) | *Ps. minutipalpata* | 3 | 0.000-0.026 | 0.139-0.148 | 0.121-0.142 | 0.146-0.148 | 0.141-0.144 | 0.126-0.137 | 0.147 | 0.143 | 0.134-0.145 | 0.143-0.147 | - |  |  |  |  |  |  |  |  |  |  |  |
| (11) | *Ps. pallidemaculata* | 1 | - | 0.158 | 0.147-0.154 | 0.166-0.170 | 0.150 | 0.156-0.166 | 0.132 | 0.153 | 0.146 | 0.133 | 0.136-0.142 | - |  |  |  |  |  |  |  |  |  |  |
| (12) | *Ps. alpina* **sp. nov.** | 1 | - | 0.172 | 0.122-0.133 | 0.135-0.143 | 0.141-0.142 | 0.135 | 0.154 | 0.143 | 0.141-0.144 | 0.148-0.152 | 0.109-0.116 | 0.147 | - |  |  |  |  |  |  |  |  |  |
| (13) | *Ps. amoena* **sp. nov.** | 3 | 0.003-0.009 | 0.190 | 0.155-0.165 | 0.139-0.143 | 0.158-0.162 | 0.150-0.160 | 0.164-0.166 | 0.133-0.135 | 0.170-0.179 | 0.136-0.146 | 0.143-0.157 | 0.177-0.181 | 0.136-0.138 | - |  |  |  |  |  |  |  |  |
| (14) | *Ps. ximalaya* **sp. nov.** | 1 | - | 0.154 | 0.137-0.145 | 0.145-0.146 | 0.154-0.158 | 0.126-0.132 | 0.131 | 0.143 | 0.118 | 0.145-0.155 | 0.143-0.149 | 0.150 | 0.147 | 0.149-0.151 | - |  |  |  |  |  |  |  |
| (15) | *Ps. zhuoma* **sp. nov.** | 2 | 0.011 | 0.178-0.180 | 0.137-0.154 | 0.143-0.150 | 0.128-0.135 | 0.141-0.145 | 0.141-0.147 | 0142-0.147 | 0.162-0.172 | 0.122-0.137 | 0.139-0.150 | 0.153-0.164 | 0.130-0.141 | 0.098-0.118 | 0.143-0.145 | - |  |  |  |  |  |  |
| (16) | *Ps. insularis* | 1 | - | 0.165 | 0.148-0.162 | 0.152-0.162 | 0.143-0.152 | 0.135-0.141 | 0.175 | 0.158 | 0.162 | 0.137-0.143 | 0.166-0.182 | 0.174 | 0.158 | 0.160-0.162 | 0.148 | 0.150-0.158 | - |  |  |  |  |  |
| (17) | *Ps. nitidifrons* | 4 | 0.008-0.020 | 0.143-0.147 | 0.129-0.138 | 0.145-0.154 | 0.143-0.156 | 0.143-0.149 | 0.164-0.168 | 0.165-0.169 | 0.156-0.160 | 0.148-0.158 | 0.145-0.152 | 0.149-0.157 | 0.144-0.152 | 0.166-0.172 | 0.143-0.147 | 0.163-0.173 | 0.157-0.163 | - |  |  |  |  |
| (18) | *Ps. silvana* | 1 | - | 0.171 | 0.152-0.162 | 0.150-0.156 | 0.149-0.151 | 0.127-0.129 | 0.153 | 0.136 | 0.145-0.149 | 0.130-0.132 | 0.139-0.149 | 0.172 | 0.160 | 0.150-0.152 | 0.138 | 0.137-0.140 | 0.132 | 0.168-0.171 | - |  |  |  |
| (19) | *Ps. amnicola***sp. nov.** | 4 | 0.008-0.060 | 0.112-0.145 | 0.124-0.154 | 0.137-0.148 | 0.126-0.135 | 0.112-0.145 | 0.145-0.152 | 0.138-0.157 | 0.155-0.164 | 0.147-0.151 | 0.126-0.147 | 0.130-0.139 | 0,146-0.150 | 0.166-0.172 | 0.141-0.153 | 0.124-0.139 | 0.134-0.141 | 0.123-0.136 | 0.141-0.151 | - |  |  |
| (20) | *Ps. mailangang* **sp. nov.** | 2 | 0.014 | 0.141-0.143 | 0.137-0.143 | 0.135-0.141 | 0.137-0.142 | 0.137-0.145 | 0.162-0.164 | 0.162-0.168 | 0.167-0.168 | 0.139-0.147 | 0.144-0.168 | 0.156-0.158 | 0.167-0.175 | 0.166-0.174 | 0.152 | 0.134-0.148 | 0.150-0.155 | 0.093-0.107 | 0.158-0.162 | 0.102-0.116 | - |  |
| (21) | *Ps. mystica* **sp. nov.** | 1 | - | 0.178 | 0.147-0.167 | 0.166-0.168 | 0.160-0.162 | 0.145-0.153 | 0.158 | 0.163 | 0.140 | 0.151-0.153 | 0.141-0.145 | 0.170 | 0.141 | 0.160 | 0.141 | 0.166-0.177 | 0.156 | 0.178-0.188 | 0.151 | 0.142-0.153 | 0.170-0.181 | - |

N, numbers of specimens of each species involved in the analysis; intra, intraspecific distances; inter, interspecific distances.
