# Supplementary material for: Integrative taxonomy of the genus Pseudostegana (Diptera, Drosophilidae) from China, with descriptions of eleven new species
Source: PeerJ. 2018 Sep 5;6:e5160. doi: 10.7717/peerj.5160 (PMC6129143; doi:10.7717/peerj.5160)
Supplement: Supplemental Information 5 [file peerj-06-5160-s005.docx]

Table S4. Summary of intra- and interspecific genetic distances of *ND2* region.

|  | Species | N | intra | inter | | | | | | | | | | | | | | | | | | | |
| --- | --- | --- | --- | --- | --- | --- | --- | --- | --- | --- | --- | --- | --- | --- | --- | --- | --- | --- | --- | --- | --- | --- | --- |
|  |  |  |  | (1) | (2) | (3) | (4) | (5) | (6) | (7) | (8) | (9) | (10) | (11) | (12) | (13) | (14) | (15) | (16) | (17) | (18) | (19) | (20) |
| (1) | *Ps. meiduo* **sp. nov.** | 1 | - | - |  |  |  |  |  |  |  |  |  |  |  |  |  |  |  |  |  |  |  |
| (2) | *Ps. xanthoptera* | 2 | 0.000 | 0.142 | - |  |  |  |  |  |  |  |  |  |  |  |  |  |  |  |  |  |  |
| (3) | *Ps. meiji* **sp. nov.** | 3 | 0.007-0.013 | 0.166-0.167 | 0.95-0.98 | - |  |  |  |  |  |  |  |  |  |  |  |  |  |  |  |  |  |
| (4) | *Ps. stictiptrata* **sp. nov.** | 2 | 0.006 | 0.156-0.158 | 0.112-0.116 | 0.122-0.130 | - |  |  |  |  |  |  |  |  |  |  |  |  |  |  |  |  |
| (5) | *Ps. stigmatptera* **sp. nov.** | 2 | 0.005 | 0.155 | 0.087-0.091 | 0.083-0.085 | 0.115-0.119 | - |  |  |  |  |  |  |  |  |  |  |  |  |  |  |  |
| (6) | *Ps. acutifoliolata* | 1 | - | 0.170 | 0.125 | 0.155-0.156 | 0.152-0.155 | 0.137-0.138 | - |  |  |  |  |  |  |  |  |  |  |  |  |  |  |
| (7) | *Ps. angustifasciata* | 2 | 0.003 | 0.183-0.184 | 0.137-0.138 | 0.159-0.162 | 0.164-0.168 | 0.137-0.141 | 0.149-0.152 | - |  |  |  |  |  |  |  |  |  |  |  |  |  |
| (8) | *Ps. bifasciata* | 2 | 0.003 | 0.175-.0176 | 0.128-0.129 | 0.157-0.160 | 0.153-0.158 | 0.141-0.142 | 0.082-0.083 | 0.153-0.156 | - |  |  |  |  |  |  |  |  |  |  |  |  |
| (9) | *Ps. bilobata* | 2 | 0.017 | 0.150-0.153 | 0.128-0.129 | 0.146-0.154 | 0.151-0.160 | 0.131-0.137 | 0.145-0.147 | 0.123-0.128 | 0.149-0.151 | - |  |  |  |  |  |  |  |  |  |  |  |
| (10) | *Ps. minutipalpata* | 2 | 0.020 | 0.176 | 0.121-0.123 | 0.168-0170 | 0.163-0.168 | 0.138-0.140 | 0.156-0.158 | 0.153-0.158 | 0.162-0.167 | 0.133-0.135 | - |  |  |  |  |  |  |  |  |  |  |
| (11) | *Ps. pallidemaculata* | 1 | - | 0.183 | 0.159 | 0.199-0.202 | 0.171-0.172 | 0.172-0.173 | 0.165 | 0.155-0.156 | 0.165 | 0.153-0.158 | 0.157-0.169 | - |  |  |  |  |  |  |  |  |  |
| (12) | *Ps. alpina* **sp. nov.** | 1 | - | 0.179 | 0.131 | 0.168-0.169 | 0.162-0.167 | 0.149-0.151 | 0.155 | 0.153-0.155 | 0.153-0.154 | 0.137-0.141 | 0.108 | 0.166 | - |  |  |  |  |  |  |  |  |
| (13) | *Ps. amoena* **sp. nov.** | 3 | 0.001-0.008 | 0.173-0.174 | 0.133-0.138 | 0.158-0.167 | 0.173-0.177 | 0.134-0.139 | 0.147-0.151 | 0.136-0.142 | 0.151-0.154 | 0.125-0.130 | 0.151-0.157 | 0.145-0.149 | 0.162-0.163 | - |  |  |  |  |  |  |  |
| (14) | *Ps. ximalaya* **sp. nov.** | 1 | - | 0.154 | 0.118 | 0.148-0.151 | 0.155-0.160 | 0.130-0.131 | 0.104 | 0.138-0.142 | 0.111-0.112 | 0.119-0.120 | 0.142-0.143 | 0.148 | 0.152 | 0.135-0.138 | - |  |  |  |  |  |  |
| (15) | *Ps. zhuoma* **sp. nov.** | 2 | 0.003 | 0.175-0.179 | 0.135-0.139 | 0.146-0.159 | 0.153-0.160 | 0.133-0.138 | 0.151-0.154 | 0.121-0.128 | 0.152-0.157 | 0.121-0.130 | 0.160-0.166 | 0.141-0.145 | 0.156-0.160 | 0.072-0.079 | 0.135-0.138 | - |  |  |  |  |  |
| (16) | *Ps. insularis* | 1 | - | 0.198 | 0.148 | 0.180-0.183 | 0.173-0.178 | 0.161 | 0.176 | 0.181 | 0.186-0.187 | 0.165-0.169 | 0.183-0.192 | 0.182 | 0.185 | 0.164-0.167 | 0.173 | 0.163-0.166 | - |  |  |  |  |
| (17) | *Ps. nitidifrons* | 4 | 0.006-0.014 | 0.161-0.165 | 0.118-0.122 | 0.134-0.142 | 0.136-0.142 | 0.129-0.132 | 0.151-0.154 | 0.155-0.160 | 0.152-0.154 | 0.153-0.161 | 0.160-0.166 | 0.167-0.171 | 0.154-0.158 | 0.156-0.161 | 0.147-0.151 | 0.138-0.146 | 0.165-0.170 | - |  |  |  |
| (18) | *Ps. silvana* | 2 | 0.007 | 0.162-0.164 | 0.131-0.132 | 0.146-0.158 | 0.136-0.142 | 0.131-0.133 | 0.152-0.155 | 0.152-0.156 | 0.151-0.153 | 0.142-0.146 | 0.165-0.170 | 0.171-0.172 | 0.169-0.171 | 0.138-0.143 | 0.138-0.139 | 0.144-0.149 | 0.123-0.127 | 0.143-0.148 | - |  |  |
| (19) | *Ps. amnicola***sp. nov.** | 4 | 0.005-0.056 | 0.176-0.190 | 0.129-0.147 | 0.157-0.176 | 0.159-0.167 | 0.139-0.160 | 0.150-0.154 | 0.155-0.171 | 0.142-0.162 | 0.152-0.165 | 0.165-0.179 | 0.175-0.180 | 10.59-0.169 | 0.153-0.171 | 0.151-0.155 | 0.151-0.166 | 0.178-0.198 | 0.069-0.092 | 0.154-0.168 | - |  |
| (20) | *Ps. mailangang* **sp. nov.** | 2 | 0.013 | 0.173-0.176 | 0.142-0.148 | 0.153-0.162 | 0.161-0.169 | 0.141-0.148 | 0.166-0.167 | 0.165-0.174 | 0.160-0.166 | 0.167-0.171 | 0.172-0.183 | 0.178-0.179 | 0.168-0.174 | 0.157-0.165 | 0.157-0.159 | 0.146-0.153 | 0.185 | 0.056-0.068 | 0.161-0.165 | 0.087-0097 | - |

N, numbers of specimens of each species involved in the analysis; intra, intraspecific distances; inter, interspecific distances.
