# Supplementary material for: Integrative taxonomy of the genus Pseudostegana (Diptera, Drosophilidae) from China, with descriptions of eleven new species
Source: PeerJ. 2018 Sep 5;6:e5160. doi: 10.7717/peerj.5160 (PMC6129143; doi:10.7717/peerj.5160)
Supplement: Supplemental Information 6 [file peerj-06-5160-s006.docx]

Table S5. Summary of intra- and interspecific genetic distances of *28S* region.

|  | Species | N | intra | inter | | | | | | | | | | | | | | | | | | | |
| --- | --- | --- | --- | --- | --- | --- | --- | --- | --- | --- | --- | --- | --- | --- | --- | --- | --- | --- | --- | --- | --- | --- | --- |
|  |  |  |  | (1) | (2) | (3) | (4) | (5) | (6) | (7) | (8) | (9) | (10) | (11) | (12) | (13) | (14) | (15) | (16) | (17) | (18) | (19) | (20) |
| (1) | *Ps. meiduo* **sp. nov.** | 1 | - | - |  |  |  |  |  |  |  |  |  |  |  |  |  |  |  |  |  |  |  |
| (2) | *Ps. xanthoptera* | 2 | 0.000 | 0.023 | - |  |  |  |  |  |  |  |  |  |  |  |  |  |  |  |  |  |  |
| (3) | *Ps. meiji* **sp. nov.** | 3 | 0.000 | 0.026 | 0.005 | - |  |  |  |  |  |  |  |  |  |  |  |  |  |  |  |  |  |
| (4) | *Ps. stictiptrata* **sp. nov.** | 2 | 0.001 | 0.023-0.024 | 0.002-0.003 | 0.002-0.003 | - |  |  |  |  |  |  |  |  |  |  |  |  |  |  |  |  |
| (5) | *Ps. stigmatptera* **sp. nov.** | 3 | 0.001 | 0.022-0.023 | 0.001-0.002 | 0.003-0.005 | 0.001-0.003 | - |  |  |  |  |  |  |  |  |  |  |  |  |  |  |  |
| (6) | *Ps. acutifoliolata* | 1 | - | 0.030 | 0.019 | 0.019 | 0.019-0.020 | 0.017-0.019 | - |  |  |  |  |  |  |  |  |  |  |  |  |  |  |
| (7) | *Ps. angustifasciata* | 2 | 0.000 | 0.023 | 0.009 | 0.009 | 0.009-0.010 | 0.008-0.009 | 0.016 | - |  |  |  |  |  |  |  |  |  |  |  |  |  |
| (8) | *Ps. bifasciata* | 2 | 0.000 | 0.028 | 0.016 | 0.016 | 0.016-0.017 | 0.015-0.016 | 0.002 | 0.014 | - |  |  |  |  |  |  |  |  |  |  |  |  |
| (9) | *Ps. bilobata* | 2 | 0.001 | 0.022-0.023 | 0.010-0.012 | 0.010-0.012 | 0.010-0.013 | 0.009-0.012 | 0.015-0.016 | 0.006-0.007 | 0.013-0.014 | - |  |  |  |  |  |  |  |  |  |  |  |
| (10) | *Ps. minutipalpata* | 3 | 0.000-0.001 | 0.023-0.024 | 0.012-0.013 | 0.012-0.013 | 0.012-0.014 | 0.010-0.013 | 0.016-0.017 | 0.007-0.008 | 0.014-0.015 | 0.006-0.008 | - |  |  |  |  |  |  |  |  |  |  |
| (11) | *Ps. pallidemaculata* | 1 | - | 0.024 | 0.015 | 0.015 | 0.015-0.016 | 0.014-0.015 | 0.022 | 0.015 | 0.020 | 0.014-0.015 | 0.013-0.014 | - |  |  |  |  |  |  |  |  |  |
| (12) | *Ps. alpina* **sp. nov.** | 1 | - | 0.023 | 0.012 | 0.012 | 0.012-0.013 | 0.010-0.012 | 0.019 | 0.009 | 0.016 | 0.006-0.007 | 0.002-0.003 | 0.013 | - |  |  |  |  |  |  |  |  |
| (13) | *Ps. amoena* **sp. nov.** | 3 | 0.001-0.005 | 0.028-0.032 | 0.016-0.020 | 0.016-0.020 | 0.016-0.021 | 0.015-0.020 | 0.026-0.028 | 0.014-0.017 | 0.023-0.026 | 0.015-0.020 | 0.016-0.020 | 0.020-0.023 | 0.019-0.022 | - |  |  |  |  |  |  |  |
| (14) | *Ps. ximalaya* **sp. nov.** | 1 | - | 0.027 | 0.017 | 0.017 | 0.017-0.019 | 0.016-0.017 | 0.010 | 0.010 | 0.008 | 0.014-0.015 | 0.015-0.016 | 0.019 | 0.017 | 0.020-0.022 | - |  |  |  |  |  |  |
| (15) | *Ps. zhuoma* **sp. nov.** | 2 | 0.000 | 0.033 | 0.021 | 0.021 | 0.021-0.022 | 0.020-0.021 | 0.033 | 0.021 | 0.030 | 0.022-0.023 | 0.023-0.024 | 0.024 | 0.023 | 0.009-0.013 | 0.027 | - |  |  |  |  |  |
| (16) | *Ps. insularis* | 1 | - | 0.029 | 0.010 | 0.010 | 0.010-0.012 | 0.009-0.010 | 0.022 | 0.010 | 0.020 | 0.012-0.013 | 0.013-0.014 | 0.019 | 0.015 | 0.015-0.019 | 0.019 | 0.022 | - |  |  |  |  |
| (17) | *Ps. nitidifrons* | 4 | 0.000-0.002 | 0.028-0.030 | 0.009-0.012 | 0.009-0.012 | 0.009-0.013 | 0.008-0.012 | 0.023-0.026 | 0.012-0.014 | 0.021-0.023 | 0.010-0.014 | 0.014-0.015 | 0.017-0.020 | 0.014-0.016 | 0.019-0.024 | 0.020-0.022 | 0.023-0.026 | 0.013-0.015 | - |  |  |  |
| (18) | *Ps. silvana* | 2 | 0.002 | 0.026-0.028 | 0.012-0.014 | 0.012-0.014 | 0.012-0.015 | 0.010-0.014 | 0.022-0.023 | 0.014-0.016 | 0.021 | 0.010-0.014 | 0.014-0.017 | 0.015-0.017 | 0.014-0.016 | 0.019-0.022 | 0.020-0.021 | 0.026 | 0.010-0.013 | 0.012-0.016 | - |  |  |
| (19) | *Ps. amnicola***sp. nov.** | 4 | 0.000-0.002 | 0.028 | 0.009 | 0.012 | 0.009-0.010 | 0.008-0.009 | 0.026 | 0.014 | 0.023 | 0.013-0014 | 0.016-0.017 | 0.020 | 0.016 | 0.021-0.024 | 0.022 | 0.026 | 0.015 | 0005-0.007 | 0.014-0.016 | - |  |
| (20) | *Ps. mailangang* **sp. nov.** | 2 | 0.000 | 0.027 | 0.008 | 0.008 | 0.008-0.009 | 0.007-0.008 | 0.023 | 0.010 | 0.020 | 0.009-0.010 | 0.013-0.014 | 0.016 | 0.013 | 0.017-0.021 | 0.019 | 0.022 | 0.012 | 0.001-0.003 | 0.010-0.013 | 0.003 | - |

N, numbers of specimens of each species involved in the analysis; intra, intraspecific distances; inter, interspecific distances.
