# Supplementary material for: Integrative taxonomy of the genus Pseudostegana (Diptera, Drosophilidae) from China, with descriptions of eleven new species
Source: PeerJ. 2018 Sep 5;6:e5160. doi: 10.7717/peerj.5160 (PMC6129143; doi:10.7717/peerj.5160)
Supplement: Supplemental Information 9 [file peerj-06-5160-s009.docx]

Table S8. The ABGD analysis result based on the *28S* data set.

| Groups | Taxon ID |
| --- | --- |
| Group 1 | *Ps. meiduo* |
| Group 2 | *Ps. stictiptrata* –1, *Ps. stictiptrata* –2,  *Ps. xanthoptera* –1, *Ps. xanthoptera* –2,  *Ps. stigmatptera* –1, *Ps. stigmatptera* –2,  *Ps. meiji* –1, *Ps. meiji* –2, *Ps. meiji* –3 |
| Group 3 | *Ps. amnicola* –1, *Ps. amnicola* –2, *Ps. amnicola* –3, *Ps. amnicola* –4,  *Ps. nifidifrons* –1, *Ps. nifidifrons* –2, *Ps. nifidifrons* –3, *Ps. nifidifrons* –4,  *Ps. mailangang* –1, *Ps. mailangang* –2 |
| Group 4 | *Ps. insularis* |
| Group 5 | *Ps. silvana* –1, *Ps. silvana* –2 |
| Group 6 | *Ps. ximalaya* |
| Group 7 | *Ps. acutifoliolata*,  *Ps. bifasciata* –1, *Ps. bifasciata* –2 |
| Group 8 | *Ps. pallidemaculata* |
| Group 9 | *Ps. minutipalpata* –1, *Ps. minutipalpata* –2, *Ps. minutipalpata* –3,  *Ps. alpina* |
| Group 10 | *Ps. amoena* –1, *Ps. amoena* –2, *Ps. amoena* –3 |
| Group 11 | *Ps. zhuoma* –1, *Ps. zhuoma* –2 |
| Group 12 | *Ps. angustifasciata* –1, *Ps. angustifasciata* –2 |
| Group 13 | *Ps. bilobata* –1, *Ps. bilobata* –2 |
